# The rice blast fungus SR protein 1 regulates alternative splicing with unique mechanisms

**DOI:** 10.1371/journal.ppat.1011036

**Published:** 2022-12-08

**Authors:** Wei Shi, Jun Yang, Deng Chen, Changfa Yin, Huixia Zhang, Xiaozhou Xu, Xiao Pan, Ruijin Wang, Liwang Fei, Mengfei Li, Linlu Qi, Vijai Bhadauria, Junfeng Liu, You-Liang Peng

**Affiliations:** 1 State Key Laboratory of Agrobiotechnology, China Agricultural University, Beijing, China; 2 MARA Key Laboratory of Pest Monitoring and Green Management, Department of Plant Pathology, College of Plant Protection, China Agricultural University, Beijing, China; 3 MARA Key Laboratory of Surveillance and Management for Plant Quarantine Pests, Department of Plant Biosecurity, College of Plant Protection, China Agricultural University, Beijing, China; University of Melbourne, AUSTRALIA

## Abstract

Serine/arginine-rich (SR) proteins are well known as splicing factors in humans, model animals and plants. However, they are largely unknown in regulating pre-mRNA splicing of filamentous fungi. Here we report that the SR protein MoSrp1 enhances and suppresses alternative splicing in a model fungal plant pathogen *Magnaporthe oryzae*. Deletion of *MoSRP1* caused multiple defects, including reduced virulence and thousands of aberrant alternative splicing events in mycelia, most of which were suppressed or enhanced intron splicing. A GUAG consensus bound by MoSrp1 was identified in more than 94% of the intron or/and proximate exons having the aberrant splicing. The dual functions of regulating alternative splicing of MoSrp1 were exemplified in enhancing and suppressing the consensus-mediated efficient splicing of the introns in *MoATF1* and *MoMTP1*, respectively, which both were important for mycelial growth, conidiation, and virulence. Interestingly, MoSrp1 had a conserved sumoylation site that was essential to nuclear localization and enhancing GUAG binding. Further, we showed that MoSrp1 interacted with a splicing factor and two components of the exon-joining complex via its N-terminal RNA recognition domain, which was required to regulate mycelial growth, development and virulence. In contrast, the C-terminus was important only for virulence and stress responses but not for mycelial growth and development. In addition, only orthologues from Pezizomycotina species could completely rescue defects of the deletion mutants. This study reveals that the fungal conserved SR protein Srp1 regulates alternative splicing in a unique manner.

## Author summary

The primary roles of serine/arginine-rich (SR) proteins in pre-mRNA splicing have been well established in humans, model animals and plants. Some studies have suggested that fungal SR proteins are also involved in pre-mRNA splicing, but direct evidence is lacking to illustrate how they regulate pre-mRNA splicing in fungi. Here we describe that a fungal conserved SR protein MoSrp1 in the model plant fungal pathogen *Magnaporthe oryzae* regulates alternative pre-mRNA splicing with unique mechanisms. MoSrp1 enhances and suppresses alternative splicing by binding to the GUAG consensus in target pre-mRNAs. The GUAG consensus *per se* enhances and suppresses the intron splicing efficiency of *MoATF1* and *MoMTP1*, both of which are important for mycelial growth, conidiation, and virulence. We also show that MoSrp1 is SUMOylatied for its nuclear localization and enhancing the GUAG binding capacity. Furthermore, we demonstrate that the N-terminal RNA recognition domain of MoSrp1 interacts with a splicing factor and two components of the exon-joining complex and is required for mycelial growth, development and virulence, and that the C-terminus was important only for virulence and stress responses but not for mycelial growth and development.

## Introduction

Serine/arginine (SR)-rich proteins are RNA-binding proteins that possess an N-terminal RNA recognition motif (RRM) domain and a C-terminal RS region enriched with a variable length of RS dipeptide repeats. The C-terminal RS region comprises about 50 amino acid residues, and animals and plants differ in the RS content, e.g., over 40% RS content in animals and over 20% RS content in plants [1–3]. SR proteins play a pivotal role in pre-mRNA splicing, which is fundamental to the expression of most eukaryotic genes and entails splice site recognition and the removal of non-coding introns from pre-mRNAs ensued by splicing of all exons (constitutive splicing [CS]) or skipping some exons while splicing (alternative splicing [AS]) [4]. Pre-mRNA splicing is catalyzed by the spliceosome, a dynamic RNA-protein complex consisting of five small nuclear RNAs (snRNAs; U1, U2, U4 through U6) and 150 distinct accessory proteins called splicing factors, including SR proteins and heterogeneous nuclear ribonucleoproteins (hnRNPs) [5,6]. In AS, SR proteins purportedly act as general activators, for they bind to exonic-splicing enhancers (ESEs) present on pre-mRNA via their RRM domain and recruit spliceosomal components (such as U1 and U2 snRNA) to the 5’ and 3’ splicing sites via their RS domain-splicing factor interactions. Other splicing factors (such as hnRNPs) bind to splicing silencers (SSs) in pre-mRNA; binding sites for both ESEs and SSs are located in close proximity on pre-mRNA, thus indicating that an interplay between activation and suppression regulates exon inclusion frequency [7,8].

The number of SR proteins vary across various kingdoms: humans (12 SR proteins; SRSF1 through SRSF12) [4]; and model organisms *Drosophila melanogaster* (8 SR proteins; Rbp1, Rbp1-like, Rsf1, XI6, Srp54, SC35, SF2 and B52) [9], *Caenorhabditis elegans* (7 SR proteins; Rsp-1 through Rsp-7) [10], *Arabidopsis thaliana* (18 SR proteins; RSZ21, RSZ22, RSZ22a, RSZ32, RSZ33, SCL28, SCL30, SCL30a, SCL33, SCL35, RS31a, SR1, Srp30, SRp34a, SR34a, SR34b, RSP31, RSP40 and RSP40) [11], *Schizosaccharomyces pombe* (2 SR proteins; Srp1 and Srp2) [12]. The first evidence verifying SR proteins as a splicing factor came from the studies showing that purified ASF/SF2 (SRSF1) and SC35 (SRSF2) could restore splicing activity of the splicing-deficient nuclear extracts [3,13–15]. The studies ensued after the discovery of the two SR proteins in humans revealed that SRSF1 and SRSF2 favor the selection of proximate 5’ and 3’ splicing sites (SS) and are required for the specific interactions of U1 snRNP with 5’SS and 3’SS and for the interaction between U2 snRNP and the branch-point sequence (BPS) in early stages of the assembly of spliceosomal components [16,17].

Extensive studies have been performed to unravel consensus sequence information on target pre-mRNAs that allows the binding of SR proteins thereon. For ten of the 12 SR proteins in humans, the binding consensus sequences have been determined; the consensus sequences for each SR protein are highly degenerated and may be distinct between studies for the same SR protein [4,18]. For example, SRSF1 prefers a purine-rich consensus, which is distributed not only in exons but also in introns in close proximity to splice sites [19]. The RRM domain or/and pseudo-RRM domain in individual SR proteins are supposed to confer the binding capability of consensus RNA sequences. Structural studies have revealed how the RRM or pseudo-RRM domains from several SR proteins recognize the RNA motifs [20–23]. Generally, it has been accepted that SR proteins in mammalian cells promote exon inclusion by binding ESEs although they may regulate pre-mRNA splicing in different mechanisms [7]. For example, the recruitment of SRSF1 and SRSF2 to their ESEs can strengthen weak splice sites to define the exon during the early steps of the splicing through interactions with U1 snRNP and U2 auxiliary factor (U2AF) [24]. SRSF2 may also enhance exon inclusion by binding to an ESE to antagonize the function of heterogenous nuclear ribonuclear proteins (hnRNPs) [25]. During later steps of the splicing, SR proteins, including SRSF1, SRSF2, and SRSF6, can enhance the recruitment of U4/U6.U5 tri-snRNP to the splice site [26]. Further, some SR proteins, such as SRSF3, can facilitate splicing by binding to intronic splicing enhancers [27]. However, some studies have indicated that SR proteins can function as splicing suppressors [24,28,29].

The functions of SR proteins can be regulated by posttranslational modifications. The most frequent modification is phosphorylation, which occurs in the RS-rich domain. For example, the subcellular localization and protein activities of SRSF1 is regulated by the phosphorylation status of the RS domain [30], which is catalyzed by serine-arginine protein kinase SRPK1 for nuclear re-import and location to nuclear speckles [31], and by Cdc2-like kinase Clk/Sty for release from nuclear speckles and recruitment to pre-mRNA for the splicing [32]. SRSF10 can be switched from a general splicing repressor to a sequence-specific splicing activator after phosphorylation [28]. In addition to phosphorylation, SR proteins can also be modified by acetylation, methylation, hydroxylation, neddylation, and ubiquitination [33–36].

SR proteins are involved in multiple biological processes with the above-mentioned molecular functions and modifications. Targeted disruption of SRSF1 and SRSF6 in human cells led to embryo lethality or cancer [37,38]. In *Drosophila*, nematode, and mice, individual SR proteins are known to be highly specific and have non-redundant roles in regulating developmental processes [39, 40]. Plants have comparatively more numbers of SR proteins than animals [41], some of which are unique to plants. A number of studies have also been performed to characterize plant SR proteins, but mainly in *A*. *thaliana* and rice [42,43]. Overexpression of some SR proteins in *A*. *thaliana* can lead to significant splicing changes in connection with plant growth and development [44,45]. In contrast, single gene null mutants of *A*. *thaliana* have been generated for 17 of the all 18 SR protein-encoding genes, but no detectable phenotype changes were observed in any of the single gene mutants, and only the *sc35-scl* quintuple mutant exhibits defects on vegetative development and flowering time, suggesting that plant SR proteins, including SC35 and SCL play redundant roles in splicing, plant growth and developments [46]. However, studies on the consensus RNA sequences bound by plant SR proteins are very limited. A recent study showed that AtSC35 from *A*. *thaliana* bind to the 5′-AGAAGA-3′ consensus [46].

Fungi have fewer SR proteins, usually two in each fungal species. Different from those in animals and plants, fungal SR proteins usually have a C-terminal RD/E-rich domain [47]. Srp1 in *S*. *pombe* was the first identified fungal SR protein, which is dispensable but is important for mRNA splicing [48]. *S*. *pombe* has the other SR protein, Srp2, that is essential and interacts with Srp1, and the interaction is modulated by protein phosphorylation catalyzed by an SR-specific kinase Dsk1 [12]. Srp2 can bind to purine-rich elements in ESEs to mediate the ESE function [49]. Slr1 is the Srp1 ortholog in the human pathogen *Candida albicans*, and affects growth, filamentation, host cell interactions, and virulence [50]. Slr1 mainly localizes to the nucleus but partly associates with translating ribosomes and binds to polyadenylated RNA [51]. FgSrp1 and FgSrp2 are the Srp1 and Srp2 orthologs of plant filamentous fungal pathogen *Fusarium graminearum*, either of which is dispensable but involved in the fungal growth, sexual reproduction, deoxynivalenol production, and virulence likely by regulating pre-mRNA splicing [52,53]. In general, although there have been some studies showing that fungal SR proteins control phenotypes likely by regulating mRNA splicing, it remains largely unknown how fungal SR proteins regulate pre-mRNA splicing except *S*. *pombe* Srp2.

In the present study, we set out to investigate mechanisms whereby MoSrp1 regulates pre-mRNA splicing in the model fungal plant pathogen *Magnaporthe oryzae*, which globally causes the devastating rice blast disease [54]. For its economic impacts, extensive and intensive studies have been performed to identify genes that are essential or important for the fungal pathogenicity [55]. However, information is very limited pertaining to the pre-mRNA splicing regulation on pathogenicity-related genes in *M*. *oryzae*. Here, we first showed that MoSrp1 is important for multiple phenotypes of *M*. *oryzae*, including virulence. Deletion of *MoSRP1* also resulted in the aberrant splicing of over 900 target mRNAs as a major defect at the vegetative hyphal stage. We then showed that MoSrp1 binds to the 5’-GUAG-3’ consensus, which was distributed in the exon and intron of hundreds of target mRNAs, including many that were indispensable for the fungal virulence, growth and/or development. We then provided solid evidence on exemplification that MoSrp1 enhanced and suppressed the consensus-mediated efficient splicing of the introns in *MoATF1* and *MoMTP1*, respectively. We further found that MoSrp1 interacted with MoRnps1 and MoThoc1, whose orthologues in humans are involved in the exon-joining complex (EJC). Interestingly, we found that sumoylation was required for functional MoSrp1, which has a KIE motif conserved only among the Srp1 orthologues in Pezizomycotina fungi. In addition, we showed that FgSrp1 but not the *S*. *pombe* Srp1 was able to rescue the phenotypic defects of the *ΔMosrp1* mutants, suggesting that Srp1 proteins in Pezizomycotina were functionally divergent from those in other fungi. Taken together, our study unraveled that the fungal SR protein SRP1 regulates mRNA splicing by unique mechanisms.

## Results

### Identification and phenotypic characterization of *MoSRP1*

**Fig 1:**
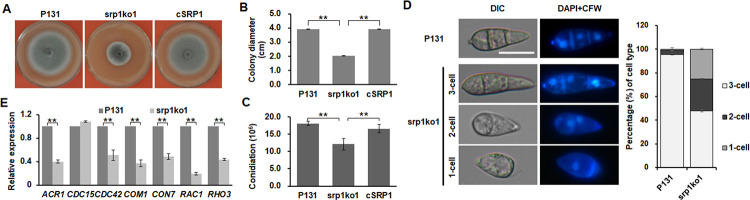
MoSrp1 is important for vegetative growth, conidia morphology, and conidiation. (A) Colonies formed by the wild-type (WT) strain P131, the Δ*Mosrp1* mutant srp1ko1, and its complementation transformant cSRP1 on OTA plates cultured for 5 d. **(B)** Statistics on colony diameters of strains P131, srp1ko1, and cSRP1 at 5 dpi. The mean and standard deviations were calculated based on three independent experiments each with three plates. Asterisk marks significant difference of the mutant from P131 and cSRP1 using *t*-test (p < 0.05). **(C)** Conidia per petri dish produced by strains P131, srp1ko1, and cSRP1. The mean and standard deviations were calculated based on three independent experiments each with three plates. Asterisk marks a significant difference of srp1ko1 from WT and cSRP1 using *t*-test (p < 0.05). **(D)** Conidia of strains P131 and srp1ko1 co-stained with CFW and DAPI. At the right of the images were percentages of conidia with 1-, 2-, and 3-cell formed by strains P131 and srp1ko1. At least 300 conidia were calculated for each strain. Bar, 10 μm. **(E)** Relative expression level of seven cell cycle-related genes in vegetative hyphae of strains P131 and srp1ko1. The gene expression level in P131 was arbitrarily set to 1. The actin gene was used as the internal control. The data were obtained from three replicates. Asterisk marks a significant difference of srp1ko1 from P131 using *t*-test (p < 0.05).

To identify the Srp1 orthologue in *M*. *oryzae*, we first performed BLASTp search against the protein database of the 70–15 and P131 strains using the *S*. *pombe* Srp1 as a query [54, 56]. The search showed that Srp1 had the highest hit with 52% identity and 55% coverage to MGG_04482 in 70–15 and P131_08116 in P131 that represented a single protein named MoSrp1. To functionally characterize *MoSRP1* in *M*. *oryzae*, two deletion mutants of the gene, srp1ko1 and srp1ko2, were generated in P131 background by the homologous recombination approach (S1 Fig). The phenotypic assay showed that vegetative mycelial growth of srp1ko1 and srp1ko2 was significantly reduced compared to that of P131 (Fig 1A and 1B). Re-introduction of the *MoSRP1-eGFP* allele into srp1ko1 generated more than 30 transformants, and all of them, including cSRP1, restored the wild-type mycelial growth (Fig 1A and 1B).

We further measured the conidium formation of the three strains. As shown in Fig 1C, the *MoSRP1* deletion mutant srp1ko1 produced 1.2×10^7^ conidia per OTA plate, which was about 60% of the wild-type P131 and the complemented cSRP1 strains. Notably, 52% of the conidia produced by srp1ko1 had less than three cells (Fig 1D). RT-qPCR analysis showed that conidium morphology-related genes were significantly down-regulated in srp1ko1, including *ACR1*, *MoCDC15*, *CDC42*, *COM1*, *CON7*, *RAC1*, and *RHO3* (Fig 1E). These results indicated that *MoSRP1* is not only important for conidiation but also involved in conidium morphogenesis.

### *MoSRP1* is required for full virulence by regulating invasive hyphal growth

**Fig 2:**
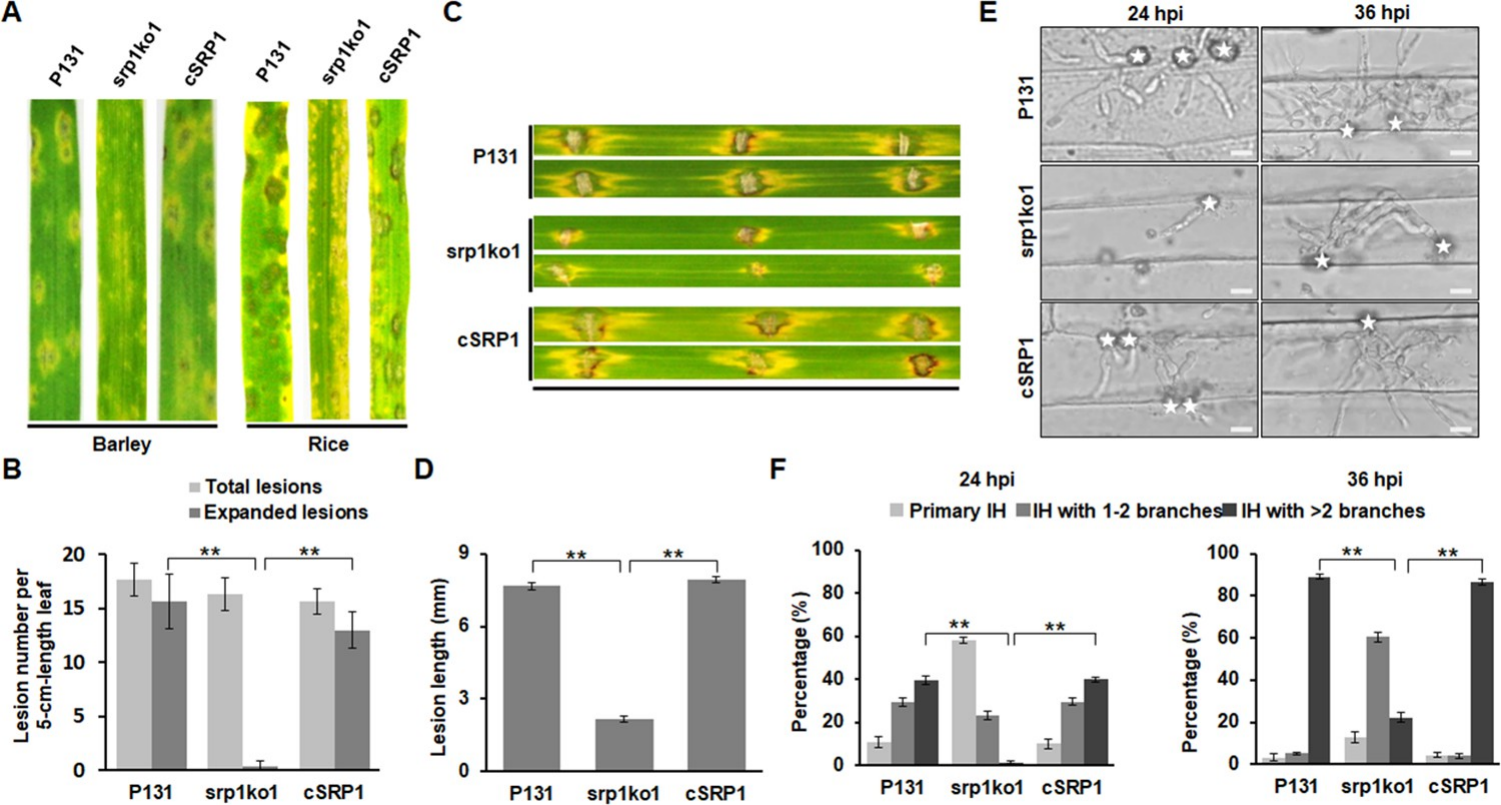
MoSrp1 is essential for full virulence. **(A)** Representative disease lesions on leaves of susceptible barley cultivar (left panel) and rice cultivar (right panel) sprayed with conidia of P131, srp1ko1, and cSRP1. **(B)** Statistics on disease lesions formed by P131, srp1ko1, and cSRP1 on susceptible rice cultivar ‘LTH’. Lesions with gray centers and larger than 2-mm in diameter were counted as expanded lesions. Means and standard deviations were calculated from the lesions formed on the middle 5-cm of the fourth leaves. Three independent experiments were conducted with at least 10 leaves in each replicate. **(C)** The top leaves of rice seedlings at the four-leaf stage were abraded and inoculated with mycelium plugs of P131, srp1ko1, and cSRP1. Representative disease symptoms were photographed at 5 dpi. **(D)** Statistics on length of individual disease lesions formed by P131, srp1ko1, and cSRP1 on abraded rice leaves as described in **(C)**. Means and standard deviations were calculated from three independent experiments, in each of which at least 10 leaves were inoculated for each of the strains. **(E)** Appressorial penetration and invasive hyphal growth of P131, srp1ko1, and cSRP1 in barley epidermis at 24 and 36 hpi. Asterisk marks appressorium. Bar, 10 μm. **(F)** Statistics on percentages of appressoria with distinct types of invasive hyphae (IH), primary IH, IH with 1–2 branches, and IH with more than 2 branches at 24 and 36 hpi. Means and standard deviations were calculated from three independent experiments each with at least 50 appressoria scored.

We also evaluated the effect of *MoSRP1* on pathogenicity by spray-inoculation. As shown in Fig 2A, srp1ko1 produced only tiny lesions on the leaves of seedlings of rice and barley. In contrast, P131 and cSRP1 formed typical susceptible lesions. Statistics analysis indicated that these strains were not significantly different in the number of total disease lesions formed on both leaves (Fig 2B), suggesting that *MoSRP1* is important for fungal proliferation in plant tissues but not for pre-penetration developments. We thereby performed further inoculation of abraded rice leaves with mycelial plugs, which showed that both P131 and cSRP1 formed larger typical disease lesions beyond the wounded sites, while srp1ko1 produced smaller lesions around the abraded sites (Fig 2C and 2D). These results indicated that *MoSRP1* is indispensable for full virulence.

To investigate how *MoSRP1* affects plant infection, we compared the infection process of srp1ko1 with that of P131 and cSRP1 on barley leaf epidermis. These three strains were indistinguishable in conidial germination, appressorium formation and penetration (S2 Fig). However, compared with P131 and cSRP1, srp1ko1 was severely impaired in developing invasive hyphae (IH) in host cells (Fig 2E**)**. At 24 hpi, srp1ko1 remained in primary IH at 60% of infection sites, whereas P131 and cSRP1 developed the secondary IH at 70% of infection sites, at more than half of which, IH formed two branches (Fig 2F). At 36 hpi, srp1ko1 developed the secondary IH with two branches at 60% infection sites but was confined to the first infected epidermal cells, while the P131 and cSRP1 IH had more than three branches and occupied the initial penetrated plant cells at almost every infection site, at some infection sites, IH expended into the neighboring plant cells (Fig 2E and 2F). These observations indicated that *MoSRP1* is important for invasive hyphal growth of *M*. *oryzae*.

### MoSrp1 interacts with some components of the exon junction complex in the nucleus

**Fig 3:**
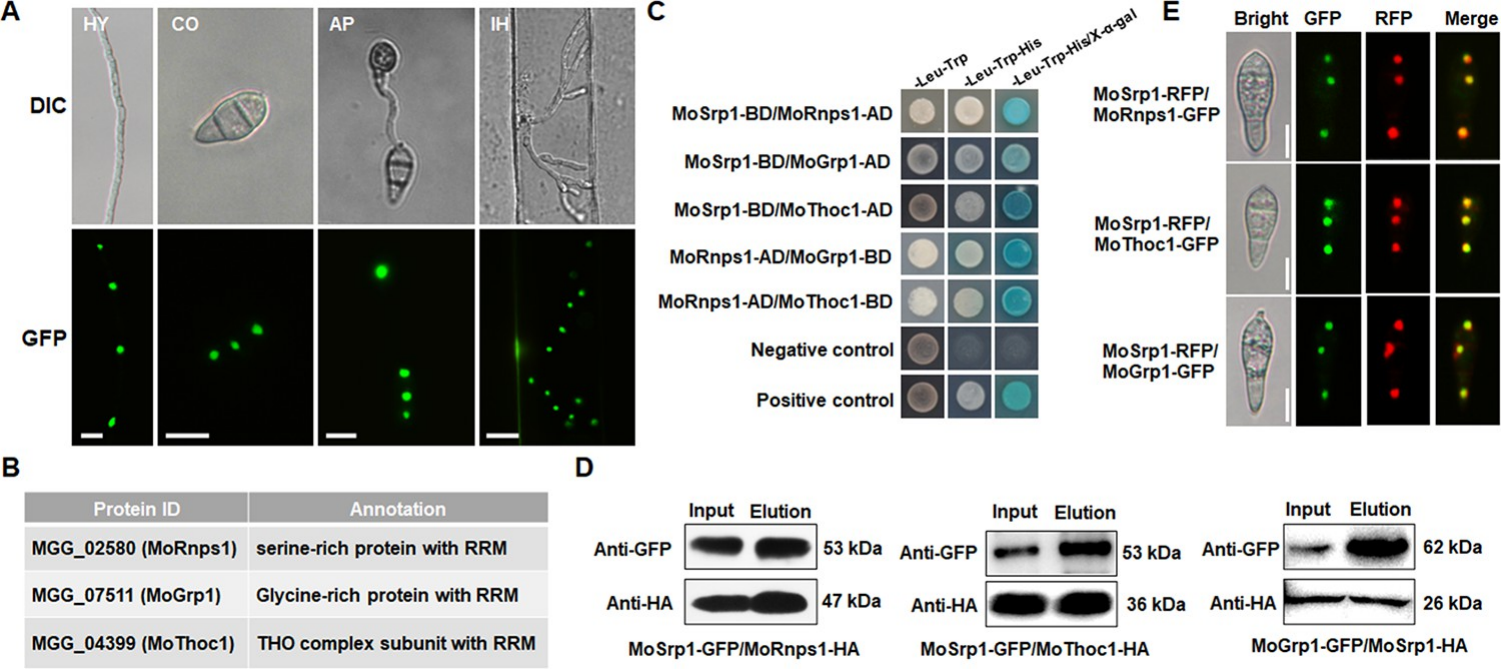
MoSrp1 is a nuclear protein and interacts with three RNA-binding proteins of the putative EJC complex. **(A)** *MoSrp1-GFP* fusion vector under the native promoter was introduced into *srp1ko1* and a transformant restored to the WT phenotypes was subsequently used to observe the GFP signals with a Nikon 90i fluorescence microscope. Photos show the images of GFP signals localized in nuclei of vegetative hyphae (HY), conidia (CO), appressorium (AP), and invasive hyphae at 24 hpi (IH24h). Bar, 10 μm. **(B)** Three MoSrp1-interacting proteins identified by immunoprecipitation and yeast library screen assays. **(C)** The yeast two-hybrid assays show interactions between MoSrp1 and MoRnps1, MoGrp1, or MoThoc1, and between MoRnps1 and MoGrp1 or MoThoc1. The interaction between AD-T and BD-Lam is used as a negative control, and the interaction between AD-T and BD-53 as a positive control. **(D)** Co-immunoprecipitation assays between MoSrp1-GFP and MoRnps1-HA or MoThoc1-HA, and between MoGrp1-GFP and MoSrp1-HA. The total and eluted proteins were immunoblotted and detected with an anti-GFP or an anti-HA antibody, and the expected sizes of hybridized bands were indicated on the right. **(E)** Co-localization of MoSrp1-RFP with MoRnps1-GFP, MoGrp1-GFP, and MoThoc1-GFP in conidia. Bar, 10 μm.

PSORTII analysis indicated that MoSrp1 has two potential nuclear localization signals at its middle region, suggesting that MoSrp1 is likely a nuclear protein. To verify the localization, we performed fluorescence microscopy with cSRP1. The GFP signals were concentrated in the nuclei of vegetative hyphae, conidia, appressoria, and infection hyphae of cSRP1 (Fig 3A). As a control, the GFP signals were even distributed in the cytoplasm of the transformants expressing *eGFP* driven by the promoter of *MoSRP1* (S3 Fig). So, MoSrp1 is a protein functioning in the nuclei.

To determine the molecular function of MoSrp1, we first identified MoSrp1-interacting proteins through IP-MS (co-immunoprecipitation coupled with mass spectrometry) and the Y2H library screening. Five putative nuclear proteins were identified among the immunoprecipitated proteins (S1 Table; Fig 3B), including MGG_02580 (named as MoRnps1), which is likely an RNA-binding protein with RRM and serine-rich domains similar to the human Rnps1, a component of the exon junction complex (EJC) [57–59]. From the Y2H library screening, eight nuclear-localized MoSrp1-interacting proteins were identified (S1 Table), and MGG_04399 and MGG_07511 were selected for further analysis (Fig 3B). MGG_04399 encodes an RNA-binding protein similar to *THOC1*, which is involved in EJC [60] and was named MoThoc1. MGG_07511 encodes a glycine-rich RNA-binding protein MoGrp1 that was recently identified as a novel splicing factor regulating virulence and growth in *M*. *oryzae* [61]. We further conducted the Y2H and Co-IP assays to validate the interactions of MoSrp1 with MoRnps1, MoThoc1, and MoGrp1. For the Y2H assays, the yeast transformants expressing each of the three combinatorial proteins grew on the selection medium, indicating that MoSrp1 physically interacts with MoRnps1, MoThoc1, and MoGrp1 (Fig 3C). Moreover, MoRnps1 physically interacted with MoThoc1 and MoGrp1 (Fig 3C). For the Co-IP assay, transformants expressing MoSrp1-GFP with either MoRnps1-HA or MoThoc1-HA were generated. As expected, in the total protein extracts and the anti-GFP affinity proteins, the 53-kD MoSrp1-GFP was detected with the 47-kD MoRnps1-HA or the 36-kD MoThoc1-HA (Fig 3D). Similarly, MoSrp1-HA was co-IPed with MoGrp1-GFP *in vivo* (Fig 3D). As a control, MoRnps1-HA or MoThoc1-HA did not interact with GFP, and MoGrp1-GFP could not interact with a non-related protein MoMas1 (S4 Fig).

We also generated transformants expressing MoSrp1-RFP fusion with MoRnps1-, MoThoc1-, or MoGrp1-GFP fusion. In conidia of the transformants, the GFP signals were clearly overlapped with the RFP signals in the nuclei, confirming that MoSrp1 is co-localized in the nucleus with MoRnps1, MoThoc1, and MoGrp1 (Fig 3E).

### MoSrp1 is an important dual-function factor enhancing or suppressing splicing

**Fig 4:**
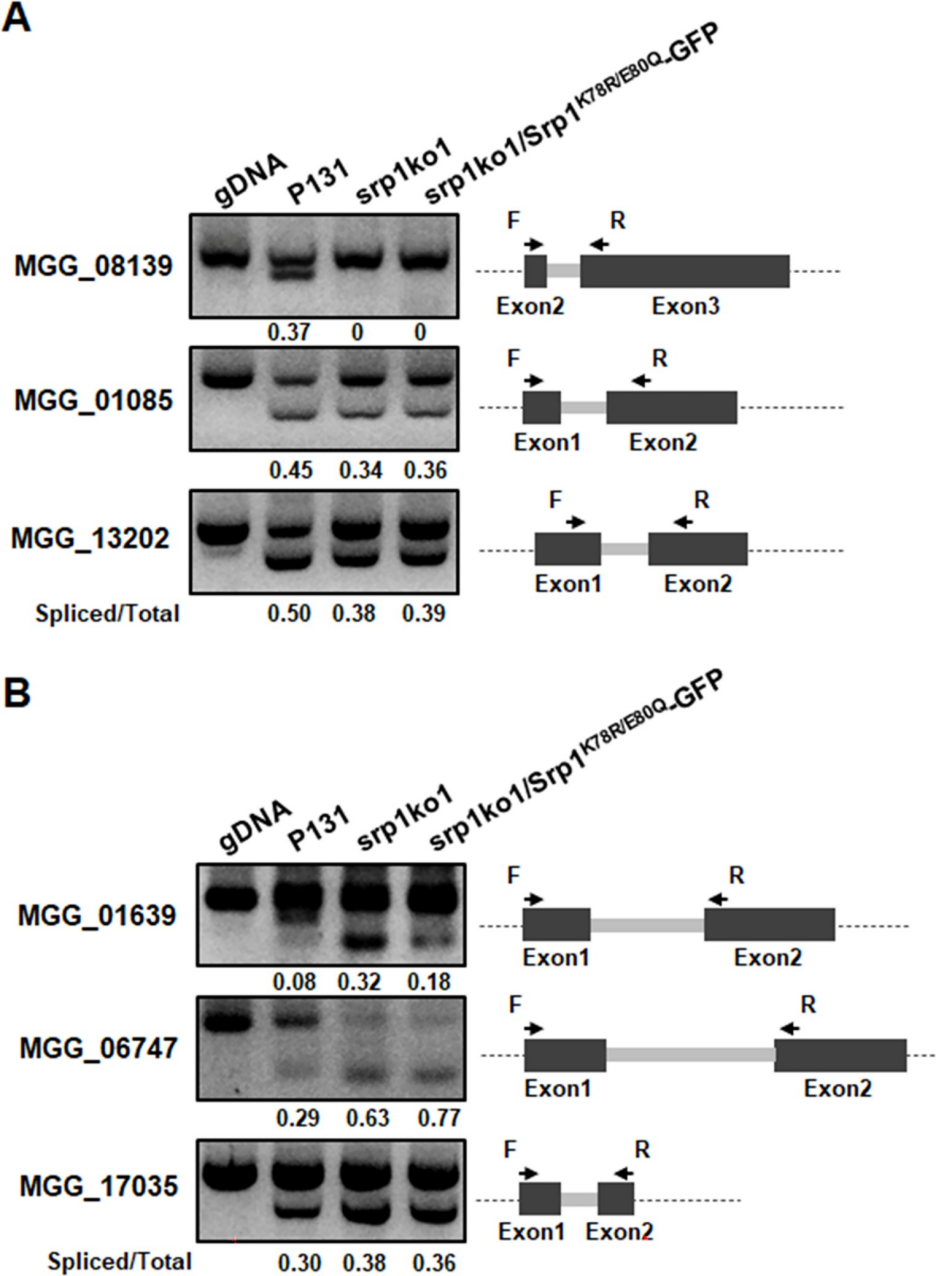
MoSrp1 regulates intron splicing efficiency. RT-PCR analyses show **(A)** enhanced splicing efficiencyand **(B)** suppressed splicing efficiency of three introns in the wild-type P131, the Δ*Mosrp1* mutant srp1ko1, and its complementation transformant cSRP1^K78R/E80Q^ expressing the mutated sumoylation site (srp1ko1/Srp1^K78R/E80Q^-GFP) allele. The corresponding genomic DNA (gDNA) fragment amplified was used as a control with the un-spliced intron. The ratio between the spliced and the total introns was shown at the bottom of each line.

Since the above-mentioned data suggested that MoSrp1 is likely a splicing factor, we investigated how MoSrp1 affects pre-mRNA splicing, first by comparing the mycelial transcriptomes of srp1ko1 and P131 and by analyzing defects in mRNA splicing resulting from the loss of *MoSRP1*. The comparative analyses revealed that the two strains expressed a total of 8,812 genes with ≥1 FPKM (Fragments Per Kilobase of exon model per Million mapped fragments) in the CM cultured mycelia, including 2,048 genes that were differentially expressed between the two strains (591 genes had Log2≥0.5, and 1457 genes had Log2≤-0.5; *p* <0.05) (S2 Table), suggesting that MoSrp1 is important for the expression of these genes. With the RNA-seq data, we further performed analysis on different splicing events between the two strains and identified 1,301 aberrant splicing events those had occurred in transcripts of 905 genes in srp1ko1 (PSI (Percent spliced in) >0.05) (S3 Table). These aberrant splicing events comprised 37 exon skipping events (ES), 88 5’-alternative splicing site events (A5SS), 110 3’-alternative splicing site events (A3SS), and 1,066 intron retention events (IR) in transcripts of 33, 78, 101 and 806 genes, respectively. To validate the bioinformatics results, we performed RT-PCR analysis on the splicing of six introns. As shown in Fig 4A, three less efficiently spliced introns revealed by the bioinformatics analyses were more retained in the transcripts of srp1ko1 than in that of P131, including one intron that was not spliced in srp1ko1. In contrast, the other three introns were more efficiently spliced in srp1ko1 than in P131 (Fig 4B). These results suggested that MoSrp1 could enhance or suppress intron splicing as a dual-function splicing factor.

### MoSrp1 binds to the 5’-GUAG-3’ consensus in RNAs

**Fig 5:**
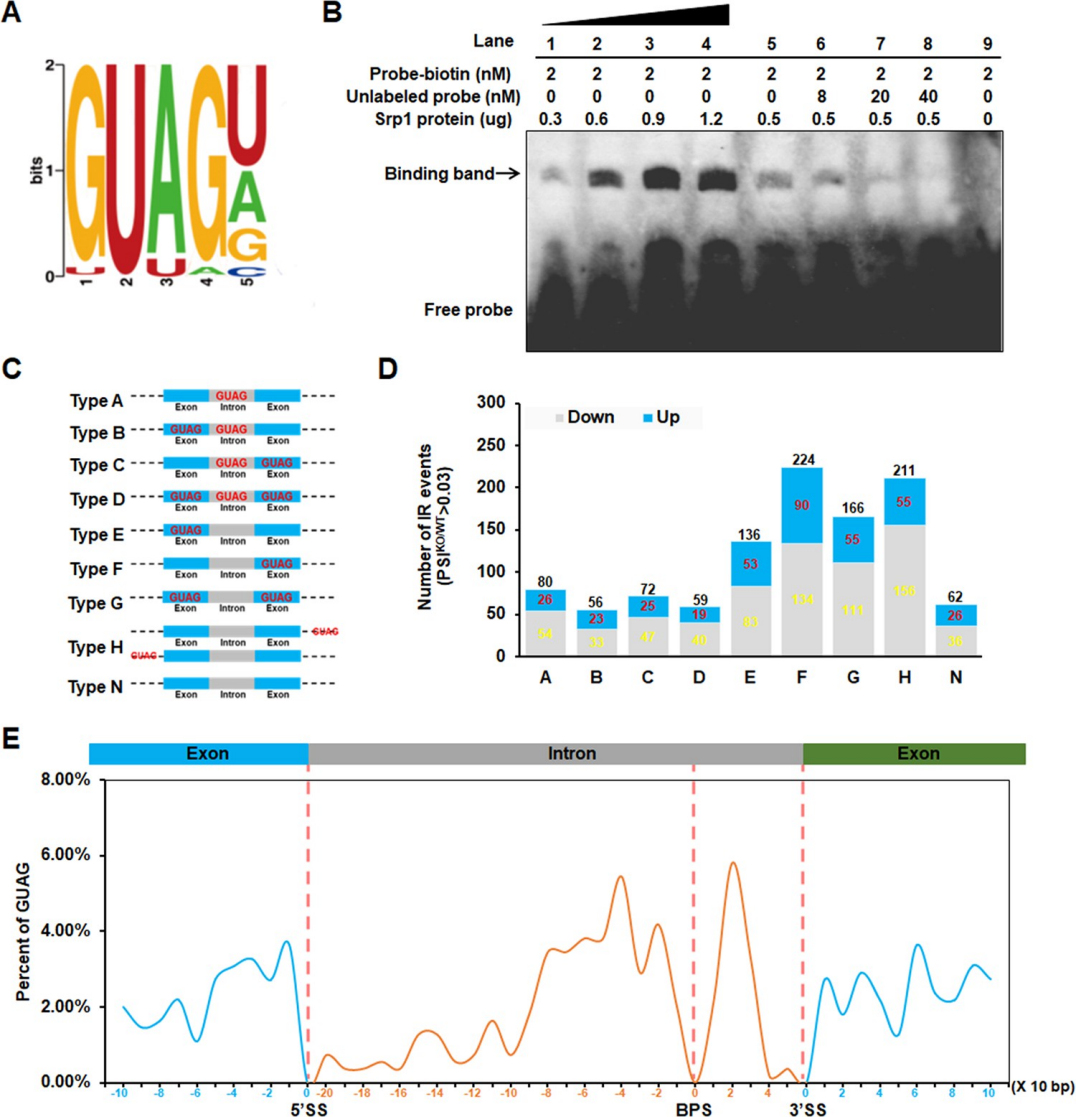
MoSrp1 binds to the GUAG consensus in pre-mRNAs. **(A)** Bit image of the top MoSrp1-binding motif by analyzing the RIP-Seq data. **(B)** RNA-EMSA assay showing the binding of MoSrp1 to the GUAG consensus. Lane 1–4: 2 nM biotin-tagged RNA fragment of 5’-GUAG-3’ with increased amount of MoSrp1 (0.3, 0.6, 0.9, and 1.2 μg); Lane 5–8: 2 nM biotin-tagged RNA fragment and 0.5 μg MoSrp1 with increased amount of unlabeled RNA oligomer (0, 8, 20, and 40 nM); Lane 9: 2 nM biotin-tagged 5’-GUAG-3’ RNA fragment without unlabeled RNA oligomer and MoSrp1. MoSrp1 was expressed and purified from *E*. *coli*, and the arrow indicates the binding band. **(C)** Different types (Type A to N) of aberrantly spliced transcripts with or without the GUAG consensus in the introns and their neighboring exons. A total of 1,066 introns retained in the Δ*Mosrp1* mutant srp1ko1 were used for analysis. **(D)** The number of intron retention events with PSI > 0.03 in srp1ko1 in comparison with WT P131. The number of introns with up- and down-regulated PSI was shown in red and yellow, respectively. **(E)** Distributions of the GUAG consensus within intron (maximum length of the MoSrp1-regulated introns is 330 nt) and its neighboring 5’- and 3’-exon with 100 nt.

To understand how MoSrp1 is involved in the regulation of alternative splicing, we identified RNA sequence bound by MoSrp1 with the RIP-seq technique by using two strains Srp1-flag-3 and Srp1-flag-7 that expressed the MoSrp1-3Flag fusion in srp1ko1. Two strains expressing 3Flag were used as a control. With the two duplicated RIP-seq assays, a short sequence, 5’-GUAG-3’ was reproducibly identified as the top consensus in the MoSrp1-bound mRNA fragments compared to the control (Fig 5A). To confirm the binding of GUAG motif by MoSrp1, RNA-EMSA assay was performed with MoSrp1-pHAT2 fusion protein expressed in *E*. *coli* (S5 Fig) and biotin-tagged RNA oligomers containing the GUAG motif. An obvious hybridization band was detected in lanes containing both MoSrp1 and the biotin-tagged RNA fragment, and was strengthened with the increased amount of MoSrp1 (Fig 5B). Moreover, the addition of unlabeled RNA oligomers with GUAG motif into the reaction mixture reduced the hybridization, more competitors were added, and fewer hybridization signals were detected. Thus, MoSrp1 was verified to directly bind to the 5’-GUAG-3’ motif in an RNA transcript.

To correlate the GUAG motif with alternative splicing, we analyzed the distribution of the GUAG motif in 905 genes that had aberrant alternative splicing events in srp1ko1. The analysis revealed that for most of the aberrant splicing events, there existed one or more GUAG motifs in a proximate position (S3 Table). Among the 1,066 introns with aberrant splicing in srp1ko1, 267 introns contained the GUAG motif, many of which also have the motifs in the proximate upstream and/or downstream exons (in 232 genes, Types A-D shown in Fig 5C and 5D and S3 Table); Surprisingly, 526 introns themselves lack the motif but in replace have a motif in their proximate upstream and/or downstream the exons (in 455 genes, Types E-G shown in Fig 5C and 5D and S3 Table), suggesting that the GUAG motif in exons may affect splicing of their proximate introns. In total, 793 of 1,066 introns had the motif in introns and/or neighboring exons (Fig 5D). Similarly, most of the exon skipping events and aberrant 5’ or 3’ splicing site events were also closely related to the existence of the GUAG motif (S3 Table). Furthermore, we analyzed the location of the GUAG motif in the introns and/or in the exons showing aberrant splicing. The frequency of the GUAG motif around the -80~30 bp of branch point sequence (BPS) is obviously higher than others, and nearly 42% of the GUAG motifs are distributed within the 100 bp upstream and 40 bp downstream of BPS in introns (Fig 5E), revealing that the GUAG motif was enriched around the branching point sites.

### MoSrp1 modulates alternative splicing at different developmental stages

**Fig 6:**
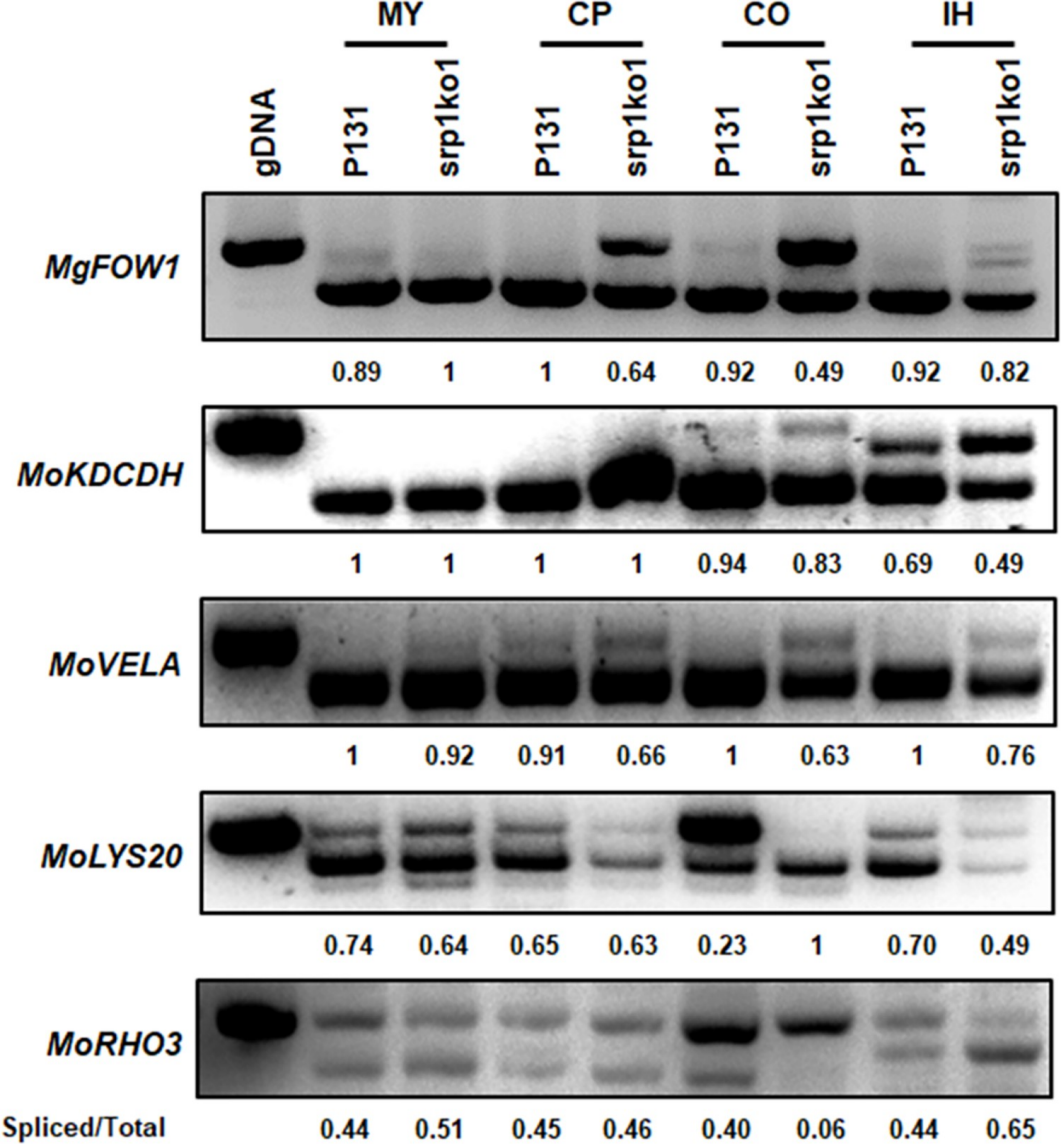
MoSrp1 regulates alternative intron splicing at different developmental stages. RT-PCR analyses show splicing efficiency of the introns of five reported genes, *MgFOW1*, *MoKDCDH*, *MoVELA*, *MoLYS20*, and *MoRHO3*, in strains P131 and srp1ko1 at different developmental stages, including mycelium (MY), conidiophores (CP), conidium (CO), and invasive hyphae (IH). The corresponding genomic DNA (gDNA) fragment amplified was used as a control with an un-spliced intron. The ratio between the spliced and the total introns was shown at the bottom of each line.

When co-analyzing the RNA-seq and RIP-seq data, we found that the MoSrp1-bound GUAG consensus was distributed in the transcripts of most genes (7,799 out of 8,812 genes) expressed in mycelia of *M*. *oryzae*, but only a small portion (905 genes) of the expressed genes displayed aberrant alternative splicing in srp1ko1. So, we questioned whether the rest genes with the GUAG consensus may have alternative splicing at other developmental stages affected by MoSrp1. To check this idea, we selected several reported infection-related genes that were not significantly different between the two strains in the alternative splicing of mycelial cells, including *MgFOW1* (MGG_07201), *MoKDCDH* (MGG_05814), *MoVELA* (MGG_08556), *MoLYS20* (MGG_01092), and *MoRHO3* (MGG_10323) [62–66] for RT-PCR analysis (Figs 6 and S6). At the conidiophore stage, the introns from *MgFOW1* and *MoVELA* were less efficiently spliced in srp1ko1. At the conidia stage, the introns from *MgFOW1*, *MoKDCDH*, *MoVELA*, and *MoRHO3* were less efficiently spliced in srp1ko1, whereas *MoLYS20* was obviously more spliced in srp1ko1. At the infection hyphae stage, the introns of *MgFOW1*, *MoKDCDH*, *MoVELA*, and *MoLYS20* were less efficiently spliced in srp1ko1; in contrast, *MoRHO3* was less efficiently spliced in P131. Taken together, the RT-PCR analyses indicated that MoSrp1 is a global splicing factor that modulates alternative splicing at growth, development, and infection stages.

### MoSrp1 enhances or suppresses intron splicing of genes important for colony growth, conidiation, and virulence

**Fig 7:**
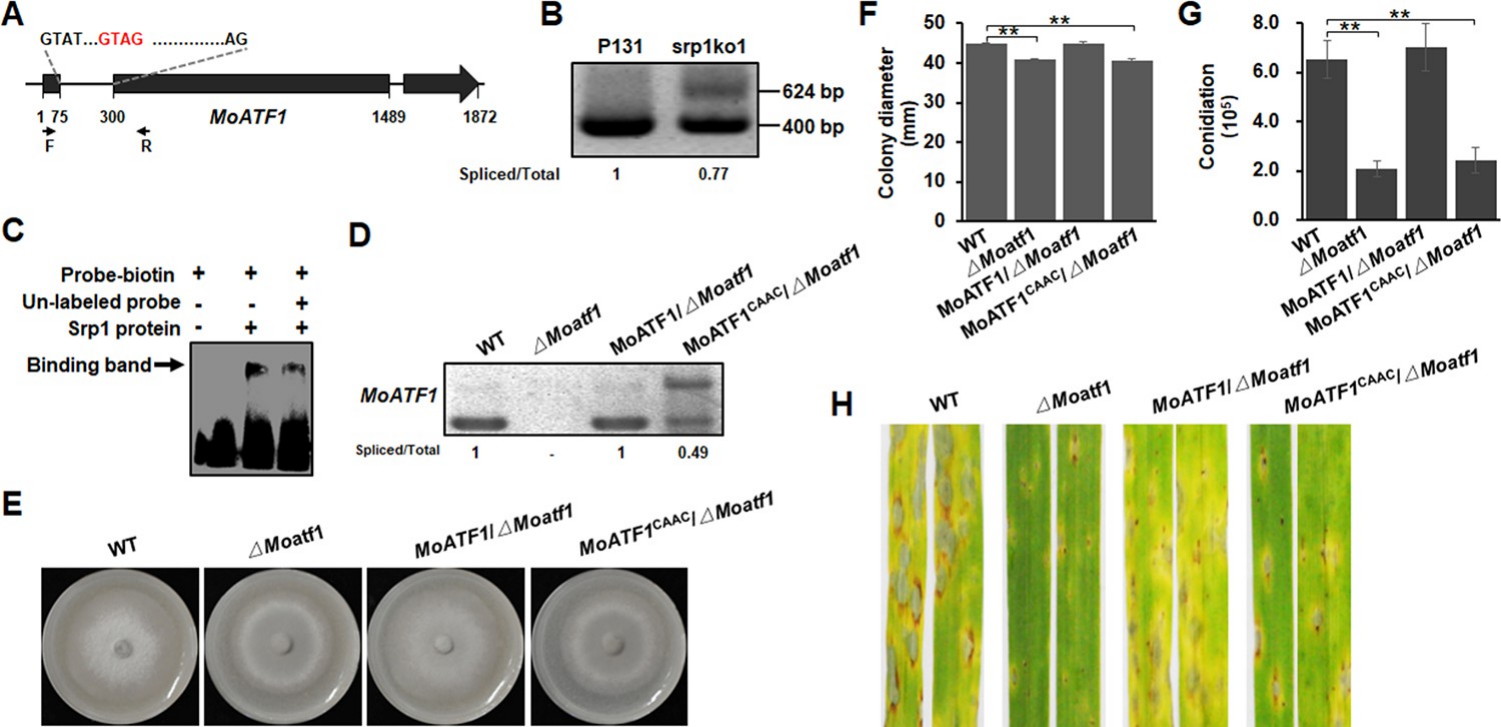
MoSrp1 enhances the intron splicing efficiency of a virulence gene *MoATF1*. **(A)** Position of the 5’-GUAG-3’ motif in the *MoATF1* pre-mRNA. **(B)** RT-PCR analysis showing splicing efficiency of the first intron of *MoATF1* in the Δ*Mosrp1* mutant srp1ko1 as compared with the wild-type P131. The ratio between the spliced and the total introns was shown at the bottom of each line. **(C)** RNA-EMSA assay showing the binding strength between MoSrp1 and the 5’-GUAG-3’ consensus in the first intron of *MoATF1*. **(D)** RT-PCR analysis showing splicing efficiency of *MoATF1* or *MoATF1*^*CAAC*^ in strains wild-type (WT) Guy11, Δ*Moatf1*, *MoATF1*/Δ*Moatf1*, and *MoATF1*^CAAC^/Δ*Moatf1* (mutation of 5’-GUAG-3’ to 5’-CAAC-3’ in *MoATF1* transcript). Guy11 was used as the wild-type strain to generate the Δ*Moatf1* mutant. The ratio between the spliced and the total introns was shown at the bottom of each line. **(E)** Colony formed by strains WT, Δ*Moatf1*, *MoATF1*/Δ*Moatf1*, and *MoATF1*^CAAC^/Δ*Moatf1* on OTA plates at 5 dpi. **(F)** Statistics on colony diameters of strains WT, Δ*Moatf1*, *MoATF1*/Δ*Moatf1*, and *MoATF1*^CAAC^/Δ*Moatf1*. **(G)** Conidia per petri dish produced by strains WT, Δ*Moatf1*, *MoATF1*/Δ*Moatf1*, and *MoATF1*^CAAC^/Δ*Moatf1* on OTA plates. The means and standard deviations in (F and G) were calculated based on three independent experiments each with three plates measured. Asterisk marks a significant difference between the Guy11 and mutant strains using *t*-test (p < 0.05). **(H)** Representative disease lesions on leaves of susceptible barley sprayed with conidia of strains WT, Δ*Moatf1*, *MoATF1*/Δ*Moatf1*, and *MoATF1*^CAAC^/Δ*Moatf1*.

To verify how MoSrp1 functions as a dual-function splicing factor to facilitate its biological roles, we checked whether any of the previously characterized genes had aberrant intron splicing in srp1ko1. Among the 905 genes that had aberrant intron splicing in srp1ko1, 68 genes have been reported to be essential or important for colony growth, conidiation or/and virulence (S4 Table), including *MoATF1* (MGG_08212) and *PPG1* (MGG_01690) [67]. *MoATF1* encodes a basic leucine zipper (bZIP) transcription factor and is important for growth, conidiation and virulence in *M*. *oryzae* [68]. In *MoATF1* pre-mRNA, the first intron with 224-bp contains a GUAG motif around 10-nt downstream of 5’SS (Fig 7A). When a pair of primers across this intron was used for RT-PCR, a 400-bp band was amplified in P131 while a bigger band with 624-bp was produced in addition to the 400-bp band in srp1ko1 (Fig 7B), showing that the intron was retained in part of *MoATF1* mRNAs in srp1ko1. Moreover, a synthesized biotin-labeled RNA fragment originated from this intron with the GUAG motif exhibiting strong binding by MoSrp1 (Fig 7C). To further determine whether the GUAG motif mediates the first intron splicing of *MoATF1*, we mutated the GUAG to CAAC in *MoATF1* and transformed this mutation allele into the *MoATF1* knockout mutant. This mutation led to the retention of the first intron in half the transcripts (Fig 7D). In contrast, the intron in P131 and a complementation transformant with the wild-type *MoATF1* allele was fully spliced from the pre-mRNAs (Fig 7D). Moreover, *MoATF1* with the mutation was dysfunctional, unable to rescue colony growth, conidiation, and plant infection although the mutant intron was removed from half of the transcripts (Fig 7E–7H). Taken together, the first intron of *MoATF1* was a direct target of MoSrp1 and its splicing efficiency was enhanced by MoSrp1, which was essential for *MoATF1* transcripts to be functional.

**Fig 8:**
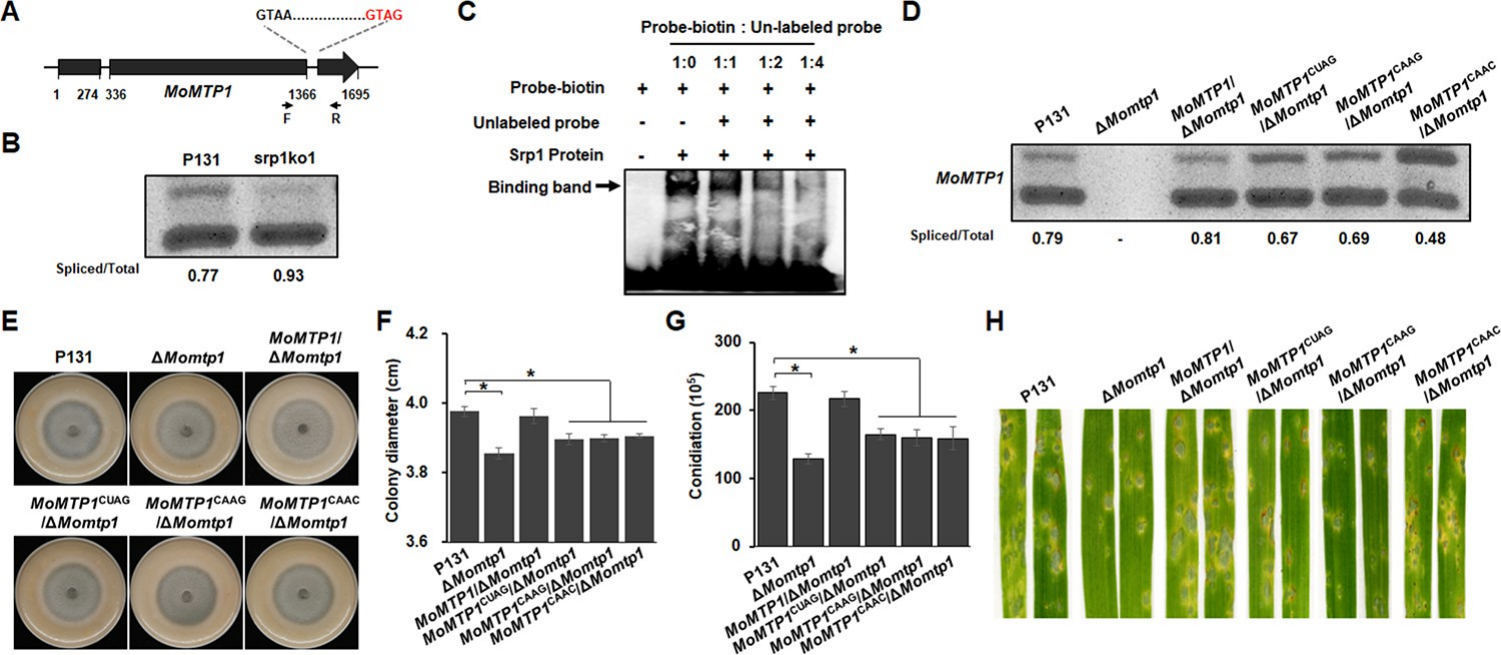
MoSrp1 suppresses the intron splicing efficiency of a novel virulence gene *MoMTP1*. **(A)** Position of the 5’-GUAG-3’ motif in the *MoMTP1* pre-mRNA. **(B)** RT-PCR analysis showing splicing efficiency of the second intron of *MoMTP1* transcript in the Δ*Mosrp1* mutant srp1ko1 as compared with the wild-type P131. The ratio between the spliced and the total introns was shown at the bottom of each line. **(C)** RNA-EMSA assay showing binding of MoSrp1 to the 5’-GUAG-3’ consensus in the *MoMTP1* intron. **(D)** RT-PCR analysis on splicing efficiency of the second intron of the *MoMTP1* transcript in strains P131, Δ*Momtp1*, and *MoMTP1*/Δ*Momtp1*, the *MoMTP1*^*CUAG*^ transcript in strain *MoMTP1*^CUAG^/Δ*Momtp1*, the *MoMTP1*^*CAAG*^ transcript in strain *MoMTP1*^CAAG^/Δ*Momtp1*, and the *MoMTP1*^*CAAC*^ transcript in strain *MoMTP1*^CAAC^/Δ*Momtp1*. The ratio between the spliced and the total introns was shown at the bottom of each line. **(E)** The colony formed by strains P131, Δ*Momtp1*, *MoMTP1*/Δ*Momtp1*, *MoMTP1*^CUAG^/Δ*Momtp1*, *MoMTP1*^CAAG^/Δ*Momtp1* and *MoMTP1*^CAAC^/Δ*Momtp1* strains on OTA plates at 5 dpi. **(F)** Statistics on colony diameters of the strains described in (E). **(G)** Conidia per petri dish produced by the strains described in (E) on OTA plates. The means and standard deviations in (F and G) were calculated based on three independent experiments each with three plates measured. Asterisk marks significant differences between P131 and mutant strains using *t*-test (p < 0.05). **(H)** Representative disease lesions on leaves of susceptible barley sprayed with conidia of the strains described in (E).

To exemplify the intron splicing negatively regulated by MoSrp1, we selected the second intron in the MGG_07843 for analysis, which is an uncharacterized gene of *M*. *oryzae* encoding a putative MFS superfamily transporter protein with a GUAG motif at its end and was named *MoMTP1* (Fig 8A). As shown in Fig 8B, about 22% of the *MoMTP1* transcript retained the intron in P131, whereas only about 7% of the intron was retained in srp1ko1. Moreover, a synthesized biotin-labeled RNA fragment originated from the second intron of *MoMTP1* with the GUAG motif was bound by MoSrp1 (Fig 8C). To further explore whether the GUAG motif is responsible for the second intron splicing of *MoMTP1*, we generated two deletion mutants of *MoMTP1*, mtp1ko1 and mtp1ko2 (S1 Fig), and phenotypic analysis showed that the Δ*Momtp1* mutant exhibited obvious defects on colony growth, conidiation, and virulence (Fig 8E–8H). Because the GUAG motif was overlapped with the 3’ splice site (3’SS) of the second intron, we generated four types of complementation transformants in the Δ*Momtp1* mutant mtp1ko1 background: one with the wild-type *MoMTP1*, and the other three with different types of mutation (CUAG, CAAG, and CAAC) in the second intron of *MoMTP1*. These three types of mutations resulted in varying degrees of retention (ranging from 33% to 52%) of the second intron in the *MoMTP1* transcripts, whereas the intron in P131 and a complementation transformant with the wild-type *MoMTP1* allele had about 20% retention rate (Fig 8D). Moreover, the complementation transformants expressing the three types of mutation alleles exhibited similar defects on colony growth, conidiation, and virulence as the Δ*Momtp1* mutant (Fig 8E–8H). Taken together, the second intron of *MoMTP1* was a direct target of MoSrp1 and its splicing efficiency was suppressed by MoSrp1.

## Functional MoSrp1 is SUMOylated

**Fig 9:**
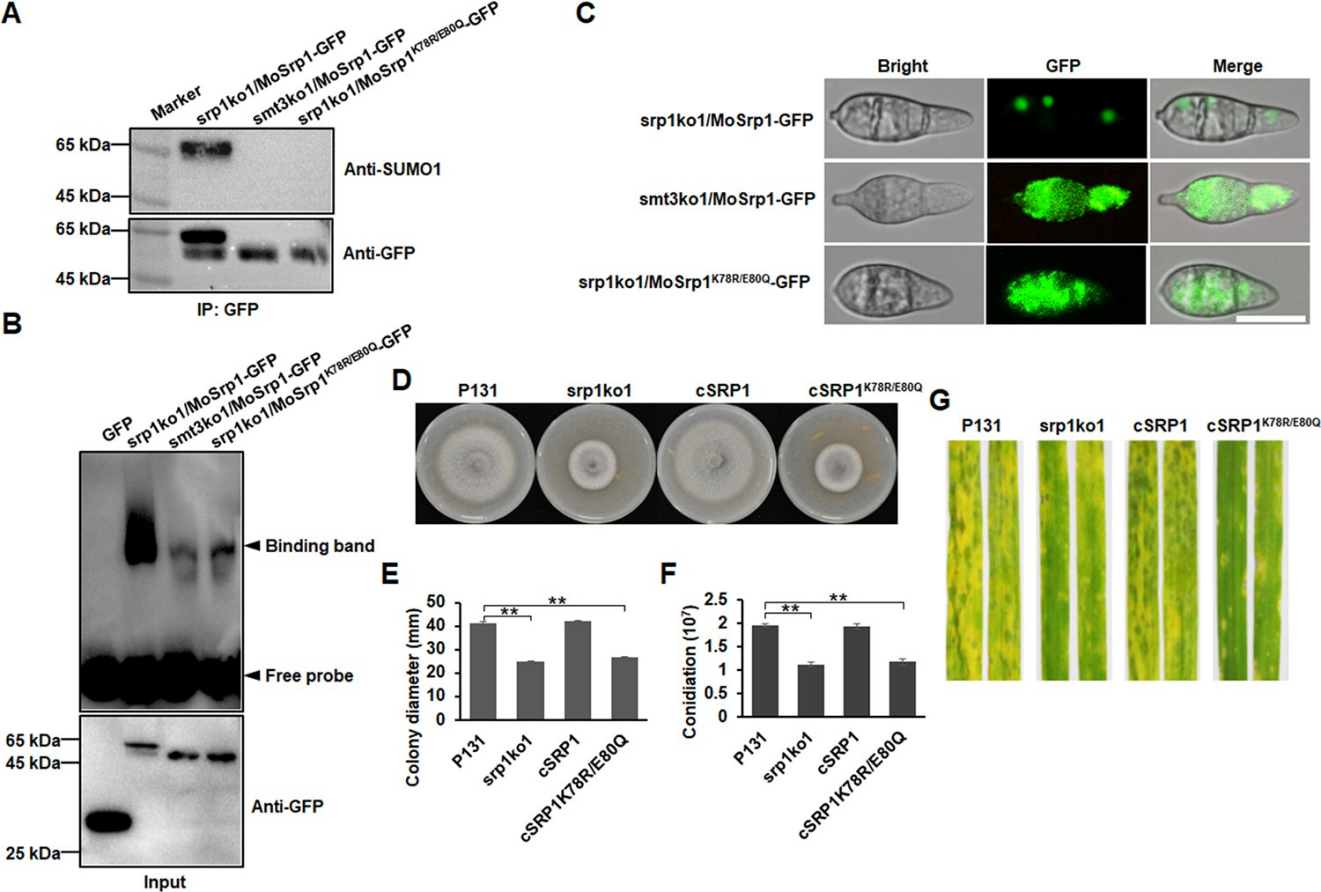
SUMOylation is required for MoSrp1 functions. **(A)** Immunoblot of the GFP-immunoprecipitated proteins from strains srp1ko1/MoSrp1-GFP, smt3ko1/MoSrp1-GFP, and srp1ko1/MoSrp1^K78R/E80Q^-GFP detected with an anti-SUMO1 antibody and an anti-GFP antibody, respectively. **(B)** RNA-EMSA assay showing binding strength of SUMOylated MoSrp1 from strain srp1ko1/MoSrp1-GFP to the 5’-GUAG-3’ consensus and the non-SUMOylated MoSrp1 from strains smt3ko1/MoSrp1-GFP and srp1ko1/MoSrp1^K78R/E80Q^-GFP (the upper panel). The proteins were detected by western blot with an anti-GFP antibody (the bottom panel). **(C)** The GFP signals are localized in the conidial nuclei of strain srp1ko1/MoSrp1-GFP while scattered in the cytoplasm of strains smt3ko1/MoSrp1-GFP and srp1ko1/MoSrp1^K78R/E80Q^-GFP. Bar, 20 μm. **(D)** Colonies formed by strains P131, srp1ko1, cSRP1, and cSRP1^K78R/E80Q^ (srp1ko1/MoSrp1^K78R/E80Q^) cultured on OTA plates for 5 d. **(E)** Statistics on colony diameters of strains P131, srp1ko1, cSRP1, and cSRP1^K78R/E80Q^. **(F)** Conidia produced by strains P131, srp1ko1, cSRP1, and cSRP1^K78R/E80Q^. The means and standard deviations in (E and F) were calculated based on three independent experiments each with three plates measured. Asterisk marks significant difference between P131 and mutant strains using *t*-test (p < 0.05). **(G)** Representative disease lesions on the leaves of susceptible barley sprayed with conidia of strains P131, srp1ko1, cSRP1, and cSRP1^K78R/E80Q^.

When assessing the expression of the MoSrp1-GFP fusion protein in srp1ko1, we detected two bands with 53-kD and 64-kD (Fig 9A). The size difference between the two bands was about 11-kD, which is a size similar to the sumoylation modification [69]. The GPS-SUMO prediction suggested that MoSrp1 has a potential sumoylation motif with K^78^IE^80^ in the RRM domain [70]. To verify whether MoSrp1 is sumoylated at this site, we expressed the MoSrp1-GFP fusion in smt3ko1, which is a mutant deleted of the SUMOylation gene *MoSMT3* [69], and the sumoylation site mutant MoSrp1^K78R/E80Q^-GFP in srp1ko1. The GFP-fusion proteins were immunoprecipitated with anti-GFP beads and were detected with anti-GFP and anti-SUMO1 antibodies. As expected, just one 53-kD band was detected for MoSrp1-GFP in smt3ko1 and MoSrp1^K78R/E80Q^-GFP in srp1ko1, whereas in srp1ko1, expressing MoSrp1-GFP, there was one bigger band in addition to the 53-kD band by an anti-GFP antibody (Fig 9A). When the anti-SUMO1 antibody was used, a 64-kD band was detected only in srp1ko1 expressing MoSrp1-GFP, but neither in smt3ko1 expressing MoSrp1-GFP nor in srp1ko1 expressing MoSrp1^K78R/E80Q^-GFP (Fig 9A). These results indicated that MoSrp1 was sumoylated at the K^78^ site by MoSmt3.

To verify whether SUMOylation affects intron splicing, we measured splicing efficiency of the first intron of *MoATF1* and the second intron of *MoMTP1* in smt3ko1. The intron splicing efficiencies of *MoATF1* and *MoMTP1* in smt3ko1 were similar to those in srp1ko1 (S7 Fig), suggesting that the SUMOylation was essential for MoSrp1 to regulate intron splicing. We then assessed the effects of the sumoylation on the RNA binding capability of MoSrp1. As shown in Fig 9B, the wild-type MoSrp1 expressed in srp1ko1 exhibited strong binding to the GUAG motif, whereas the MoSrp1 expressed in smt3ko1 and the MoSrp1^K78R/E80Q^ expressed in srp1ko1 exhibited relatively weak binding to the RNA motif, indicating that the sumoylation of MoSrp1 strengthened its RNA binding capacity. Interestingly, while the MoSrp1-GFP in srp1ko1 was localized to the nuclei, the MoSrp1-GFP in smt3ko1 and the MoSrp1^K78R/E80Q^-GFP in srp1ko1 were diffused in the cytoplasm (Fig 9C), indicating that the sumoylation was required for MoSrp1 to localize to the nuclei. Moreover, the MoSrp1^K78R/E80Q^-GFP fusion was unable to rescue srp1ko1 on colony growth, conidiation, plant infection (Fig 9D–9G), and intron splicing (Fig 4). Taken together, functional MoSrp1 requires sumoylation at the K^78^ residue.

### The RRM domain is essential to the full functions of MoSrp1 but the RD/E region is important for regulating virulence and responses to stresses

**Fig 10:**
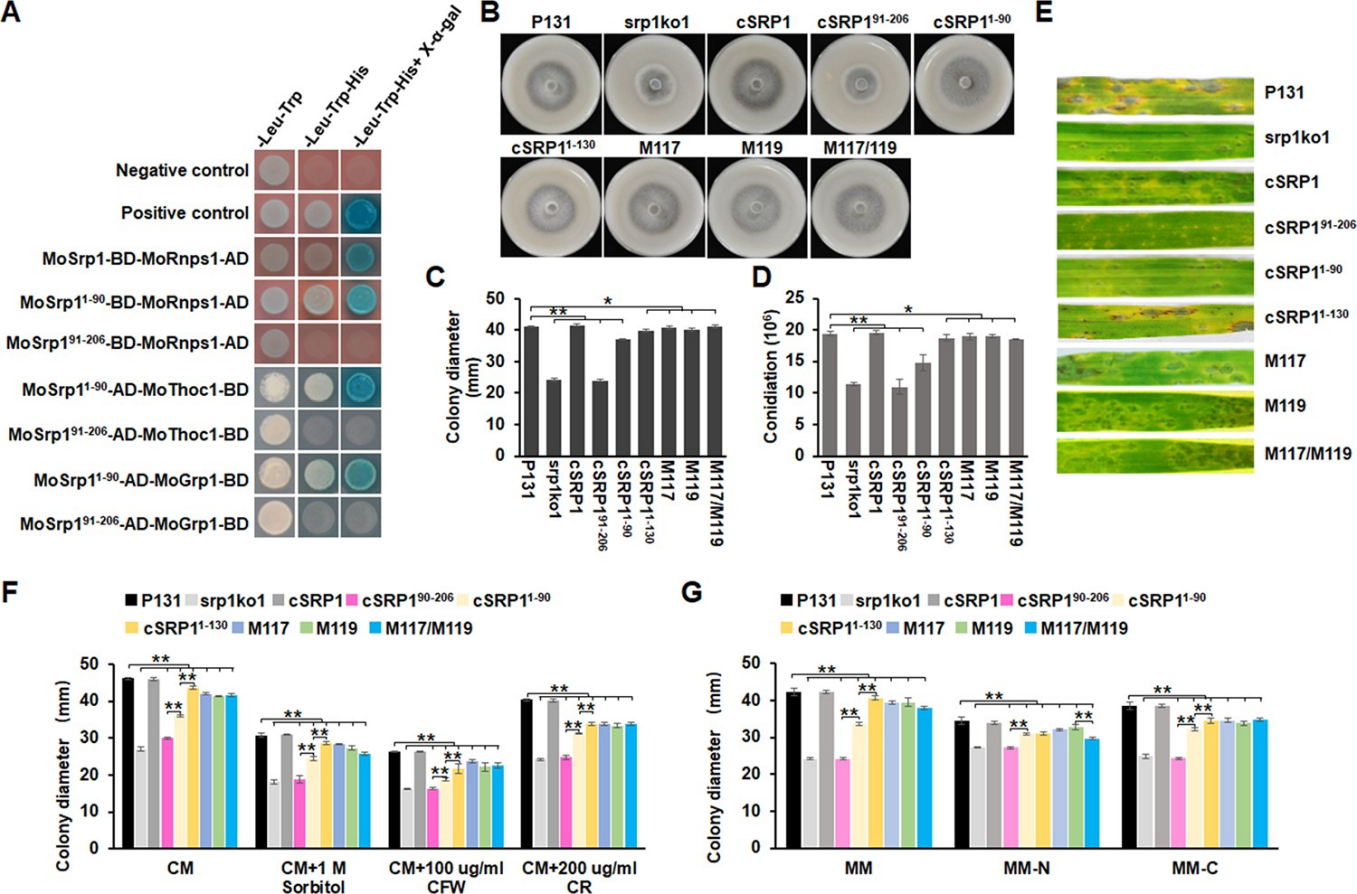
The RRM domain is essential to all the MoSrp1 functions but the SR region is important for stress responses. **(A)** The yeast two-hybrid assay showing that the interactions between N-terminus (1–90 aa) or the C-terminus (91–206 aa) of MoSrp1 and MoRnps1, MoGrp1, and MoThoc1. The interaction between AD-T and BD-Lam is used as the negative control, and the interaction between AD-T and BD-53 is used as the positive control. **(B)** The colonies formed on OTA plates at 5 dpi by the strains P131, srp1ko1, cSRP1 and srp1ko1 transformants expressing the N-terminal fragments cSRP1^1-90^ (1–90 aa), cSRP1^1-130^ (1–130 aa), the C-terminus fragment cSRP1^91-206^ (91–206 aa), mutated MoSrp1^S117A^ (M117), mutated MoSrp1^S119A^ (M119), and mutated MoSrp1^S117AS119A^ (M117/119) of MoSrp1. **(C)** Statistics on colony diameters of the strains described in (B). **(D)** Conidia per petri dish produced on OTA plates by the strains described in (B). **(E)** Representative disease lesions on the leaves of susceptible barley sprayed with conidia of the strains described in (B), and photographed at 5 dpi. **(F)** Statistical analysis of colony growth reduction rates of the strains in (B) cultured on CM plates supplemented with different stress agents (1 M sorbitol, 100 μg/ml CFW, or 200 μg/ml CR) at 5 dpi. **(G)** Statistical analysis of colony growth reduction rates of the strains described in (B) under MM, MM-N, and MM-C conditions. The means and standard deviations in (C, D, F and G) were calculated based on three independent experiments with measuring three plates for each replicate. Asterisk marks significant difference between P131 and mutant strains using *t*-test (p < 0.05).

To dissect the roles of the RRM at 7–80 aa and RD/E-rich domain at 96–172 aa, we divided MoSrp1 into two regions, MoSrp1^1-90^ with the RRM and MoSrp1^91-206^ with the RD/E-rich domain, and tested which region mediates the interaction with MoRnps1, MoThoc1, and MoGrp1 by yeast two-hybrid assay. The MoSrp1^1-90^ but not the MoSrp1^91-206^ interacted with these three proteins (Fig 10A). Then, two constructs, *MoSrp1*^*△1–90*^ and *MoSrp1*^*△91–206*^ that lacked the RRM and the RD/E-rich domain, respectively, were individually introduced into srp1ko1, and 20 transformants were randomly picked for each construct. Phenotypic assays showed that the *MoSrp1*^*△1–90*^ (cSRP1^91-206^) transformants exhibited similar mycelial growth as srp1ko1 (Figs 10B–10G; S8) while the *MoSrp1*^*△91–206*^ (cSRP1^1-90^) transformants almost restored the capacities on colony growth and conidiation on OTA plates (Fig 10B–10D). However, the cSRP1^1-90^ transformants were similar to srp1ko1 in virulence (Fig 10E), and were more sensitive than WT P131 and cSRP1 strains to chemical agents, including 1 M sorbitol, 100 μg/ml CFW, 200 μg/ml CR, MM, MM-N, and MM-C (Figs 10F–10G and S8). Therefore, the RRM domain is essential for the full functions of MoSrp1, and the RD/E-rich region is important for MoSrp1 to regulate virulence and response to stresses, albeit not important for mycelial growth and conidiation.

Based on the observations that the MoSrp1^1-90^ could not fully rescue defects of srp1ko1, we speculated that its longer sequence might fulfill its full functions and searched the conservation regions between MoSrp1 and its orthologues. The results showed that the 1–130 aa of MoSrp1 is conserved among its orthologues from Pezizomycontina fungi. So, the truncated MoSrp1^1-130^ was introduced into srp1ko1, and the resulting transformants, including cSRP1^1-130^, exhibited the wild-type phenotypes on colony growth, conidiation, and virulence (Fig 10B–10E). These findings suggested that the MoSrp1^1-130^ is enough to achieve the roles in normal growth, development and plant infection. However, the cSRP1^1-130^ transformants only partially restored tolerance to several stresses (Figs 10F–10G and S8), further indicating that the RD/E-rich region is important for regulating responses to stresses.

Previous proteomics studies showed that MoSrp1 is phosphorylated at S117 and S119 within the RD/E-rich region [71,72]. To determine whether phosphorylation at these sites affects the functions of MoSrp1, we generated constructs of *MoSRP1* with the single and double mutations of serine to alanine and introduced them into srp1ko1. Transformants with two single-site mutation alleles, M117 and M119, and the double site mutation allele, M117/M119, exhibited the wild-type phenotypes on colony growth, conidiation, and plant infection (Fig 10B–10E). However, similar to the cSRP1^1-130^ transformants, the single and double mutation alleles were obviously reduced in colony growth under stresses, including CM with 1 M sorbitol, 100 μg/ml CFW, 200 μg/ml CR, MM-N, and MM-C (Figs 10F–10G and S8). Thereby, phosphorylation of MoSrp1 within the RD/E-rich region is important for regulating stress tolerances.

### MoSrp1 orthologues in Pezizomycotina fungi are functionally similar but divergent from those in other eukaryotes

**Fig 11:**
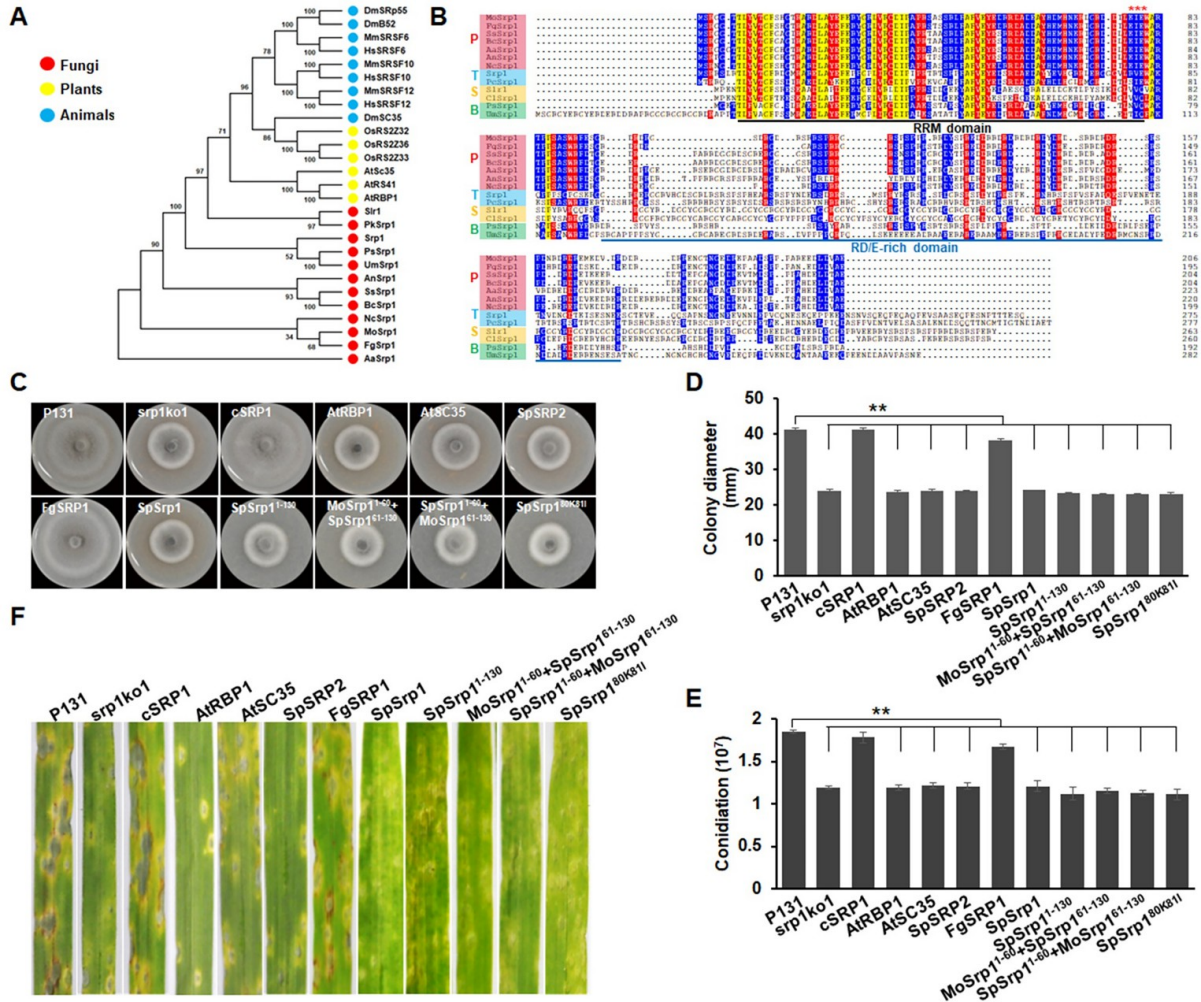
MoSrp1 homologues in Pezizomycotina fungi are functionally similar but divergent from those in other eukaryotes. **(A)** Phylogenetic analysis of MoSrp1 and its orthologues from different fungi (red dot): *Fusarium graminearum* (FgSrp1), *Sclerotinia sclerotiorum* (SsSrp1), *Botrytis cinerea* (BcSrp1), *Alternaria alternata* (AaSrp1), *Aspergillus nidulans* (AnSrp1), *Neurospora crassa* (NcSrp1), *Puccinia striformis* (PsSrp1), *Ustilago maydis* (UmSrp1), *Schizosaccharomyces pombe* (Srp1), *Pneumocystis carinii* (PcSrp1), and *Candida albicans* (Slr1), plants (yellow dot): *Arabidopsis thaliana* (AtSc35, AtRS41, and AtRBP1) and *Oryza sativa* (OsRS2Z32, OsRS2Z33, and OsRS2Z36), animals (blue dot): *Drosophila melanogaster* (DmSC35, DmSRp55, and DmB52) and *Mus musculus* (MmSRSF6, MmSRSF10, and MmSRSF12), and human *Homo sapiens* (HsSRSF6, HsSRSF10, and HsSRSF12). **(B)** Sequence alignment of MoSrp1 and its counterparts from other fungi including *Pezizomycotina* (P) FgSrp1, SsSrp1, BcSrp1, AaSrp1, AnSrp1, and NcSrp1, *Taphrinamycotina* (T) Srp1 and PcSrp1, *Saccharomycotina* (S) Slr1 and ClSrp1, and *Basidiomycota* (B) PsSrp1 and UmSrp1. The conserved amino acids among different fungi were highlighted. The RRM domain and RD/E-rich domain of MoSrp1 were underlined. An asterisk marks the SUMOylation site**. (C)** Colonies formed on OTA plates at 6 dpi by the strains P131, srp1ko1, cSRP1 and srp1ko1 transformants expressing *AtRBP1* (*AtRBP1*/srp1ko1), *AtSC35* (*AtSC35*/srp1ko1), *SpSrp2* (*SpSrp2*/srp1ko1), *SpSrp1* (*SpSrp1*/srp1ko1), *FgSrp*1 (*FgSrp1*/srp1ko1), the MoSrp1-SpSrp1 chimeric structures: SpSrp1^1-130^ (the N terminal 130 aa residues of SpSrp1), MoSrp1^1-60^-SpSrp1^61-130^ (the N terminal 60 aa residues of MoSrp1 fused with the 61–130 aa residues of SpSrp1), SpSrp1^1-60^-MoSrp1^61-130^ (the N terminal 60 aa residues of SpSrp1 fused with the 61–130 aa residues of MoSrp1), and SpSrp1^80K/81I^ (SpSrp1 mutated with the sumoylation site). **(D)** Statistics on colony growth of the strains described in (C). **(E)** Statistics on conidiation of the strains described in (C). The means and standard deviations in (D-E) were calculated based on three independent experiments by measuring three plates for each replicate. Asterisk marks significant difference between P131 and mutant strains using *t*-test (p < 0.05). **(F)** Representative disease lesions on the leaves of susceptible barley sprayed with conidia of the strains described in (C).

BLASTp searches suggested that plants and animals had three MoSrp1 orthologues, while fungi contained only one. With these MoSrp1 orthologues, we performed a phylogenetic analysis, which showed that MoSrp1 orthologues were widely distributed among eukaryotes but with high divergences (Fig 11A). Multiple sequence alignment of MoSrp1 orthologues from fungi showed that all the orthologues were similar in the RRM domain, but only those from Pezizomycotina fungi were conserved on the RD/E-rich domain (Fig 11B). Notably, MoSrp1 had the highest similarity with the orthologues in *F*. *graminearum*. Interestingly, the SUMOylation motif that was essential to MoSrp1 was conserved only in the orthologues from Pezizomycotina fungi but missed in the orthologues of fungi outside Pezizomycotina, suggesting that Srp1 orthologues may be functionally diversified even among subphylum of Ascomycota. To test this speculation, we individually introduced MoSrp1 orthologues from different species, including *F*. *graminearum* FgSrp1, *S*. *pombe* SpSrp1 and SpSrp2, and *A*. *thaliana* AtRBP1 and AtSC35 into srp1ko1. As expected, only FgSrp1 completely rescued defects of srp1ko1 on colony growth, conidiation, and plant infection (Fig 11C–11F), confirming that MoSrp1 orthologues are functionally conserved only in Pezizomycotina fungi.

To further delimit which regions of MoSrp1 result in the function diversification, we generated mosaic constructs of MoSrp1 with SpSrp1, and individually introduced them into srp1ko1 (S9 Fig). The SpSrp1^1-130^, MoSrp1^1-60^-SpSrp1^61-130^, and SpSrp1^1-60^-MoSrp1^61-130^ alleles failed to rescue defects of srp1ko1 on colony growth, conidiation, and plant infection (Fig 11C–11F). In addition, SpSrp1^80K81I^, a mutated construct generated to possess the same sumoylation site at the 80th aa as MoSrp1 also failed to rescue srp1ko1 (Fig 11C–11F). Together, these results suggested that multiple sites in the RRM domain of Srp1 proteins may be involved in functional diversification.

## Discussion

Fungal species usually carry two SR proteins Srp1 and Srp2, except the unicellular yeast *S*. *cerevisiae*, which lacks the SR proteins and alternative splicing. Different from animal and plant SR proteins that have the C-terminal RS repeats, fungal SR proteins carry RD repeats at the C-terminus [47,73]. Although a number of studies have reported that the fungal SR proteins likely function as splicing factors, it remains largely unknown how they regulate splicing. Here we provided several lines of direct evidence demonstrating that MoSrp1 in *M*. *oryzae* operates as an alternative splicing factor. First, like all previously reported SR proteins, MoSrp1 is conspicuously localized in the nucleus. Second, the deletion of *MoSRP*1 leads to >1,300 aberrant splicing events in the pre-mRNAs of 905 genes in mycelia. Third, a significant difference was detected in alternative splicing between the wild type and its *Mosrp1* deletion strains at different growth, developmental and infection stages. Fourth, MoSrp1 interacts with MoGrp1 *in vivo*, a recently identified splicing factor with the capability to bind to poly(U) in *M*. *oryzae* [61]. Fifth and importantly, MoSrp1 binds to the GUAG consensus, which is distributed in most pre-mRNAs with aberrant splicing events. Mutating the consensus therein enhanced and suppressed the splicing efficiency of the first intron in *MoATF*1 and of the second intron in *MoMTP1*, respectively, leading to reduced fungal growth, conidiation, and virulence [68]. Furthermore, we found that MoSrp1 is SUMOylated at a conserved site, which is essential to the nuclear localization and the consensus binding of MoSrp1. Therefore, the incumbent study represents the first report revealing the mechanism whereby the fungal SR protein, Srp1, modulates alternative splicing.

In animals, including mammals, SR proteins have been verified to promote alternative splicing by binding to intronic splicing enhancers (ISEs) and ESEs with their RRM domains [4,21,27,74–76]. Here we showed that the MoSrp1-binding consensus GUAG is widely distributed in target introns and/or their neighboring exons, including many introns that are more spliced in the Δ*Mosrp1* mutant than in the wild-type strain, albeit both at lesser or greater efficiencies. These findings suggest that MoSrp1 is likely a dual-function splicing factor; in many cases, it functions as a splicing enhancer, while it is a splicing suppressor in other cases. For how MoSrp1 plays dual-function roles on intron splicing, we speculate that the MoSrp1-bound GUAG consensus might be located in the proximity of binding sites for splicing enhancers or suppressors, which can recruit MoSrp1 and other spliceosomal components as a complex to promote or suppress the intron splicing. However, the detailed mechanisms remain to be explored. The dual function of MoSrp1 on regulating intron splicing is further exemplified on enhancing and suppressing the consensus-mediated efficient splicing of the introns in *MoATF1* and *MoMTP1*, respectively. Meanwhile, our study showed that the C-terminal RD/E region of MoSrp1 is not as essential as its N-terminal RRM domain to normal growth and conidiation, suggesting that MoSrp1 may bind to the GUAG consensus mainly with the RRM domain. This is different from SRSF1, which binds to an intronic splicing suppressor (ISS) through its RS region [29].

The present study showed that MoSrp1 differs from SR proteins in humans, animal, and plants in several aspects. First, MoSrp1 binds to the GUAG consensus, which is distinct from and shorter than the motifs bound by SR proteins from animals and plants. The motifs bound by animal SR proteins are usually purine-rich with 6–7 nucleotides in length [4,21,77]. The *A*. *thaliana* SR protein SC35 binds to the 5′-AGAAGA-3′ consensus motif and is also enriched with purine [46]. The shorter binding motif may allow MoSrp1 in *M*. *oryzae*, possibly SR proteins in other fungi, to regulate alternative splicing at more sites, thus decreasing the number of SR proteins in fungi. Second, sequence alignment indicated that the RRM domain of MoSrp1 is conserved only among the orthologues from Pezizomycotina fungi but divergent from that of SR proteins from animals and plants. Further complementation experiments showed that the phenotypic defects of the Δ*Mosrp1* mutants were rescued only by *FgSRP1* from the filamentous ascomycete *F*. *graminearum* [52] but not by *SRP1* from *S*. *pombe* or by *AtS35C* from *A*. *thaliana* [46,48], confirming the diversified nature of the RRM domain. Third, the C-terminal RD/E domain of MoSrp1 is not as important as the RS region of SR proteins in animals, which mediate protein-protein interactions and are required for posttranslational modifications [4]. We showed that the N-terminal 130 aa was enough to rescue all the major defects in the Δ*Mosrp1* mutant. In addition, the interactions of MoSrp1 with MoGrp1, MoRnps1, and MoThoc1 are mediated by the RRM domain but not by the C-terminal RD/E domain. Further studies will be required to determine key residues in the RRM domain of MoSrp1 that are involved in the protein-protein interaction and the binding to the GUAG consensus.

One more interesting finding in this study is that SUMOylation is essential to the functions of MoSrp1. This is the first study showing that SUMOylation regulates the functions of SR proteins. Previous studies showed that the subcellular localization and protein activities of the SR proteins in animals and plants are regulated by several types of post-translational modification (PTM) [53,78,79], which, however, did not include SUMOylation [80]. We here determined the SUMOylation at the K^78^IE site of MoSrp1, which is highly conserved in the MoSrp1 orthologues of Pezizomycotina fungi, suggesting that SUMOylation may be a conserved modification for regulating functions of Srp1 proteins in these fungi. A recent study showed that SUMOylation is crucial for colony growth, asexual development, and plant infection in *M*. *oryzae* as the mutants lacking the SUMOylation pathway genes, *Smt3*, *Aos1*, *Uba2*, *Ubc9*, and *Siz1* were atypical for these phenotypes [69]. The deletion mutants of *MoSRP1* are similar in the phenotypes to the SUMOylation pathway gene mutants, suggesting that MoSrp1 may be a key target of the SUMOylation pathway in order to regulate colony growth, asexual developments of *M*. *oryzae*, and the plant infection. Moreover, sumoylation at the K78 site of MoSrp1 is important for nuclear localization and RNA binding. Similarly, SUMOylation has been reported to promote the nuclear import of a Polo-like kinase 1 in mammals [81], and to control the binding of heterogeneous nuclear ribonucleoprotein A2B1 (hnRNPA2B1) to specific motifs in miRNAs for sorting in exosomes [82, 83]. It will be interesting to investigate how SUMOylation affects the molecular functions of these proteins.

Deletion of *MoSRP1* resulted in pleiotropic phenotypic effects on colony growth, conidiation, and virulence. These defective phenotypes can be attributed to aberrant intron splicing of multiple gene transcripts in the mutant, including 68 functionally characterized genes that play important or essential roles in colony growth, conidiation, and virulence or pathogenicity. Among them, both *MoATF1* and *MoPPG1* are important for colony growth, conidiation, and virulence [67,68]. *MoATF1* pre-mRNA has one MoSrp1-binding motif in the first intron, and mutation of this motif exhibited effects similar to the loss-of-function of MoSrp1, suggesting that MoSrp1 is a key determinant regulating the first intron splicing of *MoATF1*. Moreover, we found that MoSrp1 is also a key determinant regulating the second intron splicing of an uncharacterized gene *MoMTP1* in previous studies, which is also indispensable for colony growth, conidiation, and virulence.

Besides the regulation of intron splicing in pre-mRNAs, SR proteins also participate in multiple post-transcriptional gene regulation processes, including the formation of an exon junction complex (EJC) [4,7]. Our study also found that MoSrp1 directly interacts with MoRnps1 and MoThoc1, whose orthologues in yeasts and humans are components of the EJC [84–87], suggesting that MoSrp1 is also involved in the EJC. Previously, the EJCs were reported to multimerize with one another and with several SR proteins, such as SRSF1, SRSF3, and SRSF7 to form super-sized complexes for exon junction, in which SR proteins are super-stoichiometric to the EJC core factors [85]. How the interactions of MoSrp1 with MoRnps1 and MoThoc1 affect the EJC, and its function remain to be addressed.

## Materials and methods

### Fungal culture conditions and nucleic acid manipulations

All fungal strains (S5 Table) were cultured and maintained on oatmeal tomato agar (OTA) plates [88]. Fungal protoplast preparation and transformation, colony growth, and conidiation were performed as described [89]. To test stress responses, colony diameter was measured at five days post-inoculation (dpi) onto minimal medium (MM), MM-N, MM-C plates, and complete medium (CM) plates supplemented with 200 μg/ml Congo Red (CR), 100 μg/ml Calcofluor White (CFW), and 1 M Sorbitol. Fungal genomic DNA was extracted from vegetative mycelia with the cetyltrimethylammonium bromide (CTAB) protocol [90]. Plasmid isolation, nucleic acid extraction, DNA gel blots, enzymatic manipulation, and sequencing were performed as described [91]. Probes for DNA gel blot were labeled with a Random Primer Labeling Kit (Takara). PCR primers (S6 Table) were synthesized by Sangon. Phylogenetic and molecular evolutionary analyses were conducted using MEGA [92]. Domains were predicted by InterproScan [93].

### Plant infection and microscopy assays

The conidial suspension was adjusted to 2×10^4^ conidia/mL in a 0.025% Tween 20 and used to spray-inoculate seedlings of four-week-old susceptible rice cultivar ‘LTH’ and eight-day-old barley cultivar ‘E9’. The infected plants were incubated in a moist chamber and maintained [94]. Lesion formation was examined at 5 to 7 dpi. An infection assay with mycelial plugs on abraded leaves was performed as described [94]. 1×10^5^ conidia/mL conidia were inoculated onto cover glass slides to observe conidial morphology and inoculated onto barley leaves to observe infection process [95]. Inoculated samples were incubated in a moist chamber at 25°C, and invasive hyphal growth was examined at 24 and 36 hpi with a Nikon 90i microscope [61]. Staining on septa and nuclei of fungal conidia and hyphae was performed as described [95].

### Gene knockout, gene complementation, and subcellular localization assays

The upstream and downstream fragments of *MoSRP1* were amplified with P1/P2 and P3/P4, and cloned into pKOV21 [67], and the resulting vector was transformed into P131 protoplasts. Putative *ΔMosrp1* mutants were screened by PCR with the paired primers P5/hu and P6/hd, and then confirmed by DNA gel blot hybridization with the probe amplified by P3/P4. A split marker approach was used for generating the gene deletion mutants of *MoMTP1* [52]. Putative Δ*Momtp1* mutants were screened and confirmed by PCR with the paired primers LB-CK/hu, RB-CK/hd, and inf/inr. For gene complementation of the *ΔMosrp1* mutant, a fragment containing *MoSRP1* and its 1.5-kb promoter region was amplified with Srp1DWF/Srp1DWR and cloned into pGTN [89]. The same strategy was used to generate the GFP fusion constructs of MoSrp1 mutation alleles. For gene complementation of the *ΔMoatf1* or *ΔMomtp1* mutant, a fragment containing *MoATF1* or *MoMTP1* and its 1.5-kb promoter region was amplified with ATF1HB-F/ATF1HB-R or MTP1HB-F/MTP1HB-R and cloned into pKN [89]. The same strategy was used to generate the MoAtf1^CAAC^ and MoMtp1^CAAC^ mutation alleles. For heterologous complementation, the cDNA fragments containing *FgSRP1*, *SpSRP1*, *SpSRP2*, *AtRBP1*, and *AtSC35* were individually cloned into pKNRG. The same strategy was used to generate the SpSrp1^1-130^, MoSrp1^1-60^+SpSrp1^61-130^, SpSrp1^1-60^+MoSrp1^1-130^, and SpSrp1^80K/81I^ alleles. The resulting constructs were transformed into a *ΔMosrp1* mutant. The *MoTHOC1*-*eGFP*, *MoGRP1-eGFP* and *MoRNPS1-eGFP* constructs and MoSrp1-RFP were co-transformed into P131. All neomycin-resistant transformants were confirmed by PCR analysis and screened for fluorescence expression under a Nikon 90i microscope.

### Yeast two-hybrid (Y2H) assays

For Y2H library screening, the pGBKT7-MoSrp1 as bait was transformed into *S*. *cerevisiae* Y2H Gold strain with the MATCHMAKER Gal4 Two-Hybrid System (Clontech). After checking protein expression and no self-activation, correct yeast strain was used for mating with the yeast library constructed from the cDNA of *M*. *oryzae* vegetative mycelia. The positive colonies can grow under the SD medium lacking Histidine (SD-L/W/H), and PCR products of the positive colonies were selected for sequencing. Different bait and prey constructs were generated by cloning the full-length cDNA or truncated alleles of *MoSRP1*, *MoTHOC1*, *MoGRP1*, and *MoRNPS1* into pGBKT7 and pGADT7, respectively. The paired resulting bait and prey constructs were co-transformed into the Gold strain, and the Trp^+^Leu^+^ transformants were selected and were tested their growth on SD/Trp^-^/Leu^-^/His^-^ medium. Yeast strains harboring the pairs of pGBKT7-53/pGADT7-T and pGBKT7-Lam/pGADT7-T were used as positive and negative controls, respectively.

### Coimmunoprecipitation (Co-IP) assays

For identifying MoSrp1 co-immunoprecipitated proteins, the *MoSRP1*-3Flag constructs were introduced into strain srp1ko1. Total proteins were extracted from the resulting transformants and incubated with anti-Flag M2 beads (Sigma), and then proteins bound to the beads were eluted for LC-MS/MS analysis. For protein-protein interaction, the *MoSRP1-*eGFP and *MoRNPS1-*HA constructs were co-introduced into strain P131, the total proteins were extracted from the resulting transformant and then incubated with GFP-Trap beads (Chromotek). Proteins bound to GFP-Trap beads were eluted according to the protocol, and western blots of the total proteins and the elution from GFP-Trap beads were performed with an anti-GFP antibody and an anti-HA antibody, respectively (Sigma). The same approach was used to assay the interaction between MoSrp1-eGFP and MoThoc1-HA, MoGrp1-eGFP and MoSrp1-HA, MoGrp1-eGFP and MoThoc1-HA, and MoGrp1-eGFP and MoRnps1-HA.

### RT-PCR and quantitative RT-PCR (RT-qPCR)

Total RNAs were extracted from vegetative hyphae grown in liquid CM, conidiophores and conidia formed on OTA plate, and invasive hyphae in barley leaves, pretreated with DNase I and then reverse transcribed with a PrimeScript RT-PCR Kit (Takara). RT-PCR was performed to evaluate the intron splicing efficiency of individual transcripts between P131 and the *ΔMosrp1* mutant. RT-qPCR was performed on an ABI 7500 real-time PCR system (Applied Biosystems) according to the manufacturer’s instructions. The actin gene (MGG_03982) was used as a control. Relative abundance of gene expression level was calculated using the 2^-ΔΔCt^ method as described previously [96].

### RNA-seq and RNA-immunoprecipitation and sequencing (RIP-seq) assays

RNA extract, high-throughput cDNA libraries construction, and comparative transcriptomic analysis were performed as described [61,95] with mycelia grown in liquid CM for two days. The transcriptomes were firstly compared with the annotated genes in the reference genome of 70–15, and then differences in exon skipping, alternative 3’SS selection, alternative 5’SS selection, and intron retention were examined between P131 and the *ΔMosrp1* mutant. Alternative splicing events were detected and quantified by using SplAdder [97]. For each event in each gene, percent-spliced-in (PSI) values were computed based on the quantified splicing graphs. The PSI values between strains P131 and srp1ko1 were compared. The events, whose PSI maximum value (> 0.03) of WT was higher than the minimum value of mutant, or otherwise, were filtered.

RNA-immunoprecipitation and cDNA library construction were performed as described [98] with the abovementioned CM-cultured mycelia. Two independent strains expressing the MoSrp1-3Flag fusion protein (Srp1-flag-3 and Srp1-flag-7) were compared with two control strains expressing the empty vector (RP27-flag-1 and RP27-flag-2), respectively. Two grams of mycelia from each sample were harvested for protein extraction, and anti-Flag beads were used to immunoprecipitate the MoSrp1-3Flag protein. The resulting complexes (beads, antibody, MoSrp1-3Flag, and RNAs) were then collected by using a magnet. Further, total RNAs from the complexes were isolated by using the phenol-chloroform-isoamyl alcohol extraction method. cDNA was then synthesized with reverse transcriptase (SuperScript IV Reverse transcriptase) following the manufacturer’s protocol. A “no reverse transcription” control (no SuperScript IV added) was prepared for each sample. The resulting cDNA was used immediately for library preparation of high-throughput sequencing. For high-throughput sequencing, the cDNA libraries were applied to the Illumina Hiseq2000 system for 150 nt paired-end sequencing by ABLife company. The RIP-seq data analysis was performed as described [99]. Two replicates were performed for both the RNA-seq and RIP-seq assays.

### RNA electrophoretic mobility shift (RNA-EMSA) assays

The pHAT2-MoSrp1 construct with the N-terminal 6His was generated and expressed in *Escherichia coli* BL21(DE3). The induced *E*. *coli* cells were harvested and lysed by sonication. Supernatants from the cell lysates were applied to a Ni-Chelating Sepharose Fast Flow column and after washing with lysis buffer, His-tagged proteins were eluted with elution buffer. The eluted proteins were then purified by gel filtration chromatography on a Superdex 75 10/300GL column (GE Healthcare). The MoSrp1-GFP and MoSrp1^K78R/E80Q^-GFP protein were extracted from P131 and incubated with GFP-Trap beads (Chromotek). Proteins bound to GFP-Trap beads were eluted according to the manual provided by the manufacturer. The protein-RNA binding assays were performed with the Light Shit Chemiluminescent RNA EMSA Kit according to the manual provided by manufacturer (Thermo Pierce).

## Supporting information

S1 Fig Gene deletion of *MoSRP1* and *MoMTP1*. **(A)** The *MoSRP1* gene knockout vector was constructed by amplifying the upstream and the downstream flanking sequences and ligated with the *hph* cassette. A, *Apa*I. **(B)** Southern blot analysis of *Apa*I-digested genomic DNAs from the wild-type P131 (lane 1) and its two Δ*Mosrp1* mutants (lane 2 and 3) hybridized with the probe in (A). The estimated size of each band is indicated at right. **(C)** A split marker approach was used for generating the gene deletion mutants of *MoMTP1*. The resistant marker gene *hph* was split into two fragments, HY and YG. The upstream and the downstream flanking sequences of *MoMTP1* were fused with the split *hph*, respectively. **(D)** PCR validated the two Δ*Momtp1* mutants, mtp1ko1 and mtp1ko2 by using the paired primers, LB-CK/hu, RB-CK/hd, and inf/inr for amplifying the LBC, RBC, and gene fragments.
(TIF)

S2 Fig *MoSRP1* is dispensable for conidial germination, appressorium formation and penetration. **(A)** Microscope observation of conidial germination (upper panel, 2 hpi on cover glass slide), appressoria formation (middle panel, 8 hpi on cover glass slide), and primary invasive hyphae (lower panel, 20 hpi on barley epidermis) by the wild-type strain P131, the Δ*Mosrp1* mutant srp1ko1, and its complementation transformant cSRP1. Bar, 20 μm. **(B)** Statistical analyses on rates of conidial germination, appressorium formation, and appressorial penetration of strains P131, srp1ko1, and cSRP1. The mean and standard deviations were calculated based on two independent experiments (n = 100 conidia or appressoria/replicate). Asterisk marks a significant difference between the mutant from P131 and cSRP1 using *t*-test (p < 0.05). *ns*, no significance.
(TIF)

S3 Fig Subcellular localization of GFP driven by the promoter of *MoSRP1* during the fungal development and plant infection. Photos show the images of GFP signals localized in the cytoplasm of vegetative hyphae (HY), conidia (CO), appressorium (AP), and invasive hyphae (IH). Bar, 10 μm.
(TIF)

S4 Fig The negative control for co-immunoprecipitation assays between MoSrp1 and its interacting proteins. Co-immunoprecipitation assays between GFP and MoRnps1-HA or MoThoc1-HA, and between MoGrp1-GFP and its non-interacting protein MoMas1-HA. The total and eluted proteins were immunoblotted and detected with an anti-GFP or an anti-HA antibody, respectively, and the expected sizes of hybridized bands were indicated on the right.
(TIF)

S5 Fig Expression and purification of MoSrp1 from *E*. *coli*. Lane M, molecular mass markers; Lane–IPTG, total protein extracted before IPTG induction; Lane +IPTG, total protein extracted after IPTG induction; Lane Total Protein, supernatant of total protein extracted after centrifugation; Lane Purified, MoSrp1 protein after affinity chromatography and gel filtration. Lane Anti-His, immunoblotting confirmation of purified MoSrp1 by an anti-His antibody.
(TIF)

S6 Fig The negative control for RT-PCR of five reported genes from different developmental stages. The total RNAs of strains P131 and srp1ko1 at different developmental stages, including mycelium (MY), conidiophores (CP), conidium (CO), and invasive hyphae (IH), were extracted and used as the template after removing the genomic DNA (gDNA) for PCR of five reported genes, *MgFOW1*, *MoKDCDH*, *MoVELA*, *MoLYS20*, and *MoRHO3*. The corresponding genomic DNA (gDNA) fragment amplified was used as a control.
(TIF)

S7 Fig Intron splicing efficiency of *MoATF1* and *MoMTP1* in the Δ*Mosmt3* mutant. RT-PCR analyses show the splicing efficiency of **(A)** the first intron of *MoATF1* and **(B)** the second intron of *MoMTP1*in the Δ*Mosmt3* mutant smt3ko1 in comparison with the wild-type P131 and the Δ*Mosrp1* mutant. The ratio between the spliced intron and the total one was shown at the bottom of each line.
(TIF)

S8 Fig The RRM domain and phosphorylation sites of MoSrp1 are important for cell wall integrity and stress tolerance. **(A)** Colony of the wild-type P131, the Δ*Mosrp1* mutant srp1ko1, and its one complementation transformant cSRP1, srp1ko1 expressing the MoSrp1 N-terminus (1–90 aa) (cSRP1^1-90^), (1–130 aa) (cSRP1^1-130^), and its C-terminus (91–206 aa) (cSRP1^91-206^), srp1ko1 expressing mutated MoSrp1^S117A^ (M117), mutated MoSrp1^S119A^ (M119), and mutated MoSrp1^S117AS119A^ (M117/119) cultured on CM plates supplemented with different stress agents including 1 M sorbitol, 100 μg/ml CFW, and 200 μg/ml CR at 5 dpi. **(B)** Colony of the strains in (A) cultured on MM, MM-N, and MM-C plates at 5 dpi.
(TIF)

S9 Fig Scheme on region dissection of MoSrp1 and SpSrp1. MoSrp1, the full-length of MoSrp1; MoSrp1^1-130^, the 1–130 aa of MoSrp1; SpSrp1, the full-length of SpSrp1; SpSrp1^1-130^, the 1–130 aa of SpSrp1; MoSrp1^1-60^-SpSrp1^61-130^, the mosaic fusion between MoSrp1^1-60^ and SpSrp1^61-130^; SpSrp1^1-60^-MoSrp1^61-130^, the mosaic fusion between SpSrp1^1-60^ and MoSrp1^61-130^; SpSrp1^80K/81I^ with mutated sumoylation sites on 80K/81I in SpSrp1.
(TIF)

S1 Table List of MoSrp1-interacting proteins identified by immunoprecipitation and yeast library screening assays. (DOCX)

S2 Table List of differential expressed genes with the deletion of *MoSRP1*. (XLSX)

S3 Table List of defects on intron splicing with the deletion of *MoSRP1*. (XLSX)

S4 Table Previously characterized genes with aberrant intron splicing in the deletion mutants of *MoSRP1*. (DOCX)

S5 Table Fungal strains used in this study. (DOCX)

S6 Table PCR primers used in this study. (XLSX)
