# Supplementary figures and images for: The rice blast fungus SR protein 1 regulates alternative splicing with unique mechanisms
Source: PLoS Pathog. 2022 Dec 8;18(12):e1011036. doi: 10.1371/journal.ppat.1011036 (PMC9767378; doi:10.1371/journal.ppat.1011036)

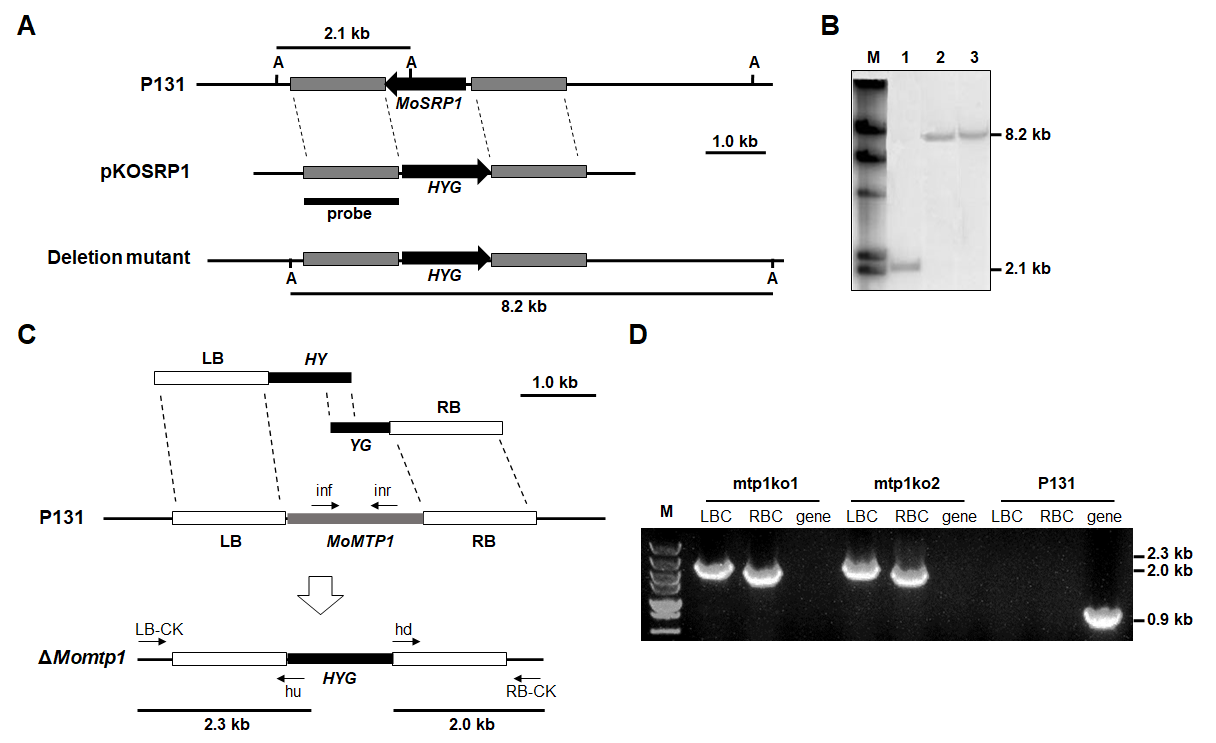

Supplement: S1 Fig — (A) The MoSRP1 gene knockout vector was constructed by amplifying the upstream and the downstream flanking sequences and ligated with the hph cassette. A, ApaI. (B) Southern blot analysis of ApaI-digested genomic DNAs from the wild-type P131 (lane 1) and its two ΔMosrp1 mutants (lane 2 and 3) hybridized with the probe in (A). The estimated size of each band is indicated at right. (C) A split marker approach was used for generating the gene deletion mutants of MoMTP1. The resistant marker gene hph was split into two fragments, HY and YG. The upstream and the downstream flanking sequences of MoMTP1 were fused with the split hph, respectively. (D) PCR validated the two ΔMomtp1 mutants, mtp1ko1 and mtp1ko2 by using the paired primers, LB-CK/hu, RB-CK/hd, and inf/inr for amplifying the LBC, RBC, and gene fragments. (TIF) [file ppat.1011036.s001.tif]

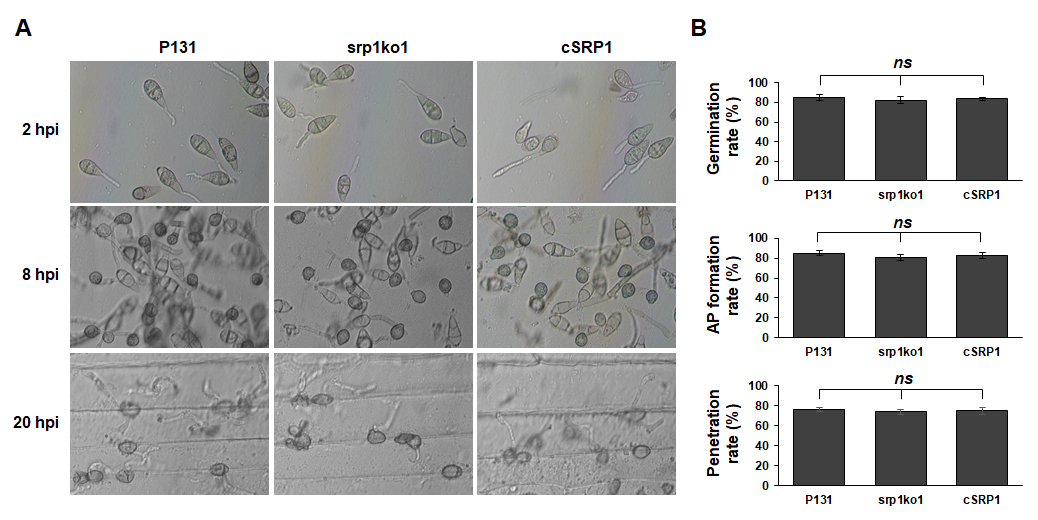

Supplement: S2 Fig — (A) Microscope observation of conidial germination (upper panel, 2 hpi on cover glass slide), appressoria formation (middle panel, 8 hpi on cover glass slide), and primary invasive hyphae (lower panel, 20 hpi on barley epidermis) by the wild-type strain P131, the ΔMosrp1 mutant srp1ko1, and its complementation transformant cSRP1. Bar, 20 μm. (B) Statistical analyses on rates of conidial germination, appressorium formation, and appressorial penetration of strains P131, srp1ko1, and cSRP1. The mean and standard deviations were calculated based on two independent experiments (n = 100 conidia or appressoria/replicate). Asterisk marks a significant difference between the mutant from P131 and cSRP1 using t-test (p < 0.05). ns, no significance. (TIF) [file ppat.1011036.s002.tif]

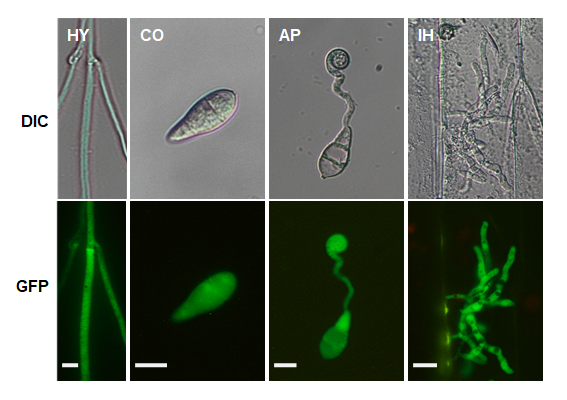

Supplement: S3 Fig — Photos show the images of GFP signals localized in the cytoplasm of vegetative hyphae (HY), conidia (CO), appressorium (AP), and invasive hyphae (IH). Bar, 10 μm. (TIF) [file ppat.1011036.s003.tif]

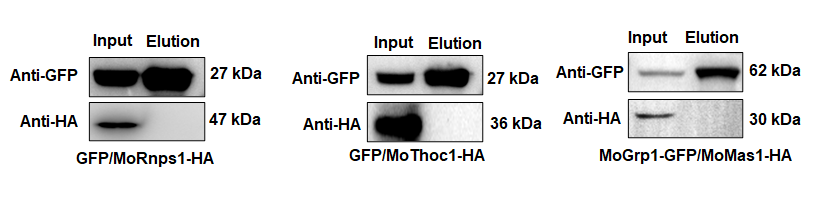

Supplement: S4 Fig — Co-immunoprecipitation assays between GFP and MoRnps1-HA or MoThoc1-HA, and between MoGrp1-GFP and its non-interacting protein MoMas1-HA. The total and eluted proteins were immunoblotted and detected with an anti-GFP or an anti-HA antibody, respectively, and the expected sizes of hybridized bands were indicated on the right. (TIF) [file ppat.1011036.s004.tif]

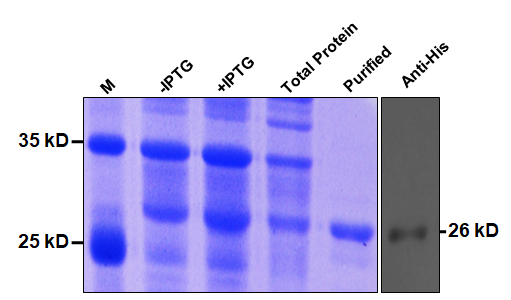

Supplement: S5 Fig — Lane M, molecular mass markers; Lane–IPTG, total protein extracted before IPTG induction; Lane +IPTG, total protein extracted after IPTG induction; Lane Total Protein, supernatant of total protein extracted after centrifugation; Lane Purified, MoSrp1 protein after affinity chromatography and gel filtration. Lane Anti-His, immunoblotting confirmation of purified MoSrp1 by an anti-His antibody. (TIF) [file ppat.1011036.s005.tif]

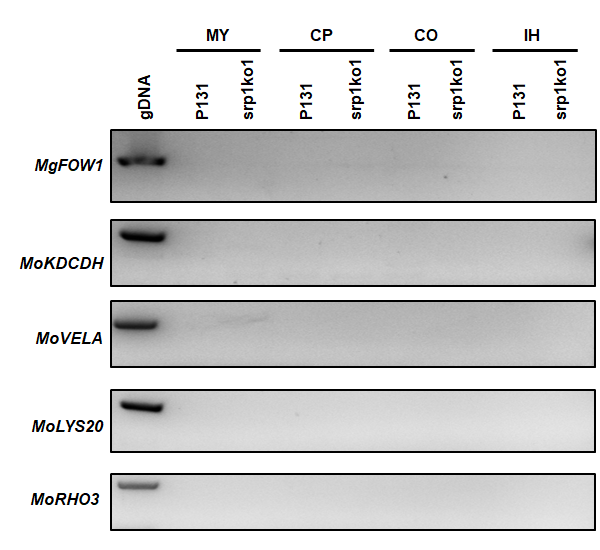

Supplement: S6 Fig — The total RNAs of strains P131 and srp1ko1 at different developmental stages, including mycelium (MY), conidiophores (CP), conidium (CO), and invasive hyphae (IH), were extracted and used as the template after removing the genomic DNA (gDNA) for PCR of five reported genes, MgFOW1, MoKDCDH, MoVELA, MoLYS20, and MoRHO3. The corresponding genomic DNA (gDNA) fragment amplified was used as a control. (TIF) [file ppat.1011036.s006.tif]

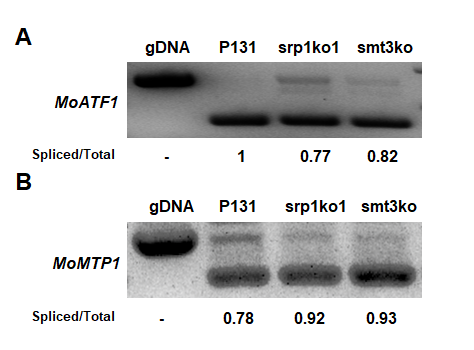

Supplement: S7 Fig — RT-PCR analyses show the splicing efficiency of (A) the first intron of MoATF1 and (B) the second intron of MoMTP1in the ΔMosmt3 mutant smt3ko1 in comparison with the wild-type P131 and the ΔMosrp1 mutant. The ratio between the spliced intron and the total one was shown at the bottom of each line. (TIF) [file ppat.1011036.s007.tif]

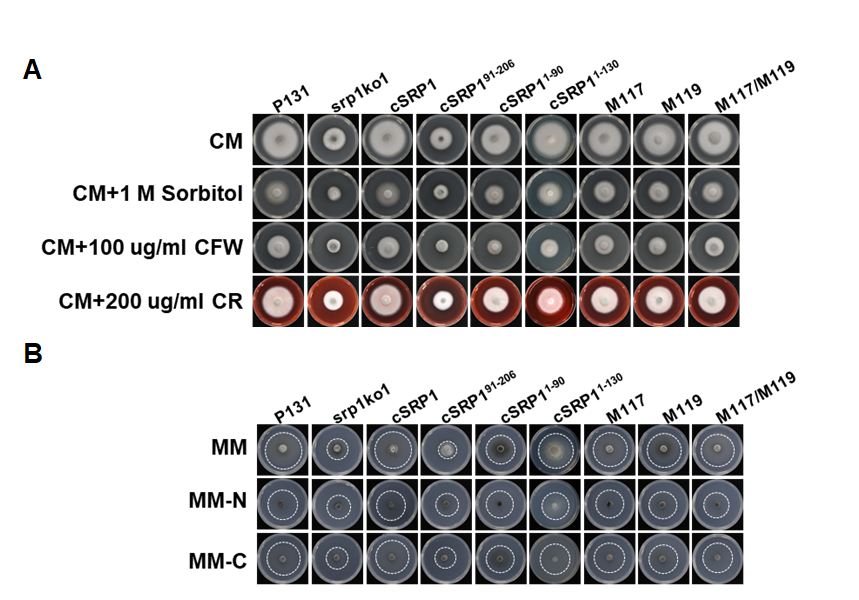

Supplement: S8 Fig — (A) Colony of the wild-type P131, the ΔMosrp1 mutant srp1ko1, and its one complementation transformant cSRP1, srp1ko1 expressing the MoSrp1 N-terminus (1–90 aa) (cSRP11-90), (1–130 aa) (cSRP11-130), and its C-terminus (91–206 aa) (cSRP191-206), srp1ko1 expressing mutated MoSrp1S117A (M117), mutated MoSrp1S119A (M119), and mutated MoSrp1S117AS119A (M117/119) cultured on CM plates supplemented with different stress agents including 1 M sorbitol, 100 μg/ml CFW, and 200 μg/ml CR at 5 dpi. (B) Colony of the strains in (A) cultured on MM, MM-N, and MM-C plates at 5 dpi. (TIF) [file ppat.1011036.s008.tif]

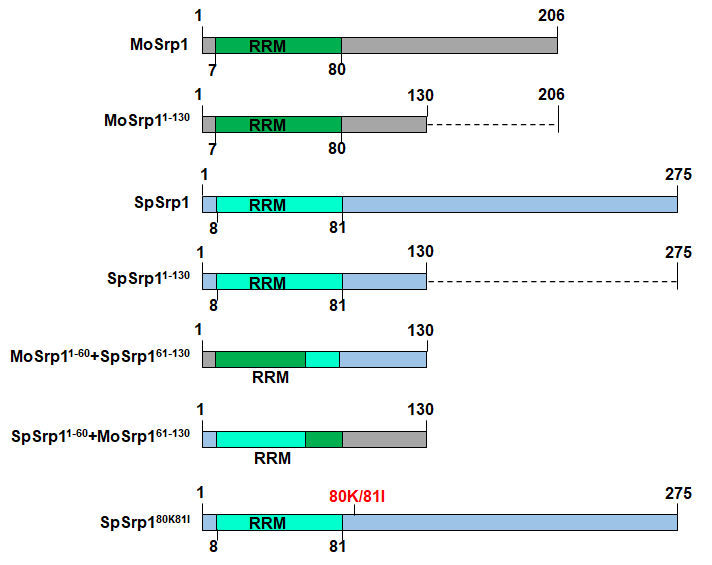

Supplement: S9 Fig — MoSrp1, the full-length of MoSrp1; MoSrp11-130, the 1–130 aa of MoSrp1; SpSrp1, the full-length of SpSrp1; SpSrp11-130, the 1–130 aa of SpSrp1; MoSrp11-60-SpSrp161-130, the mosaic fusion between MoSrp11-60 and SpSrp161-130; SpSrp11-60-MoSrp161-130, the mosaic fusion between SpSrp11-60 and MoSrp161-130; SpSrp180K/81I with mutated sumoylation sites on 80K/81I in SpSrp1. (TIF) [file ppat.1011036.s009.tif]
