# Supplementary material for: The rice blast fungus SR protein 1 regulates alternative splicing with unique mechanisms
Source: PLoS Pathog. 2022 Dec 8;18(12):e1011036. doi: 10.1371/journal.ppat.1011036 (PMC9767378; doi:10.1371/journal.ppat.1011036)
Supplement: S1 Table — (DOCX) [file ppat.1011036.s010.docx]

| **S1 Table. MoSrp1-interacting nuclear proteins identified by immunoprecipitation (IP) and yeast two-hybrid (Y2H) assays.** | | |
| --- | --- | --- |
| **Protein ID** | **Annotation** | **Approach** |
| MGG_03242 | heat shock factor Hsf24 | Y2H |
| MGG_07511 | glycine-rich protein with RRM, MoGrp1 | Y2H |
| MGG_00110 | carbohydrate esterase family 3 protein | Y2H |
| MGG_06928 | CMGC/CDK protein kinase | Y2H |
| MGG_04399 | THO complex subunit 1 with RRM, MoThoc1 | Y2H |
| MGG_06044 | ubiquitin-60S ribosomal protein L40 | Y2H |
| MGG_00814 | hsp90-like protein | Y2H |
| MGG_06760 | CAMK protein kinase, MoCAMK1 | Y2H |
| MGG_01842 | SAP domain-containing protein with RRM | IP |
| MGG_02580 | serine-rich protein with RRM, MoRnps1 | IP |
| MGG_04589 | chromodomain helicase hrp3 | IP |
| MGG_07518 | H/ACA ribonucleoprotein complex subunit 4 | IP |
| MGG_16901 | ATP-dependent RNA helicase Dbp2 | IP |
