# Supplementary material for: The rice blast fungus SR protein 1 regulates alternative splicing with unique mechanisms
Source: PLoS Pathog. 2022 Dec 8;18(12):e1011036. doi: 10.1371/journal.ppat.1011036 (PMC9767378; doi:10.1371/journal.ppat.1011036)
Supplement: S4 Table — (DOCX) [file ppat.1011036.s013.docx]

**S4 Table. Previously characterized genes with aberrant intron splicing in the deletion mutants of *MoSRP1*.**

| **Gene ID** | **Gene name** | **Colony** | **Conidiation** | **Virulence** | **Reference** |
| --- | --- | --- | --- | --- | --- |
| MGG_00329 | FZC27 | wild-type | wild-type | wild-type | [1] |
| MGG_00800 | MST7 | wild-type | wild-type | 0 | [2] |
| MGG_00883 | MCK1 | wild-type | reduced | 0 | [3] |
| MGG_00987 | ABL1 | reduced | reduced | reduced | [4] |
| MGG_01294 | MoAgo3 | reduced | reduced | reduced | [5] |
| MGG_01569 | MoYcp4 | reduced | increased | reduced | [6] |
| MGG_01661 | Mokmt3 | reduced | reduced | reduced | [7] |
| MGG_01669 | MoAOS1 | reduced | reduced | reduced | [8] |
| MGG_01690 | MoPPG1 | reduced | reduced | 0 | [9] |
| MGG_01760 | EXO70 | wild-type | wild-type | reduced | [10] |
| MGG_02006 | MobZIP04 | wild-type | increased | reduced | [11] |
| MGG_02252 | MoBUF1 | wild-type | wild-type | 0 | [12] |
| MGG_02595 | FZC7 | wild-type | wild-type | wild-type | [1] |
| MGG_02632 | MobZIP05 | wild-type | increased | reduced | [11] |
| MGG_02731 | Rac1 | wild-type | reduced | 0 | [13] |
| MGG_03148 | TDG4 | wild-type | reduced | reduced | [14] |
| MGG_03208 | Gel5 | wild-type | wild-type | wild-type | [15] |
| MGG_03463 | FZC10 | wild-type | wild-type | wild-type | [1] |
| MGG_03580 | ATG4 | wild-type | wild-type | 0 | [16] |
| MGG_04100 | SEP1 | wild-type | reduced | reduced | [17] |
| MGG_04141 | FZC69 | reduced | wild-type | wild-type | [1] |
| MGG_04236 | MoTrx2 | reduced | reduced | reduced | [18] |
| MGG_04421 | MoSNT2 | reduced | wild-type | reduced | [19] |
| MGG_04708 | MoSOM1 | reduced | 0 | 0 | [20] |
| MGG_04719 | MoMip11 | reduced | reduced | reduced | [21] |
| MGG_04895 | ICL1 | wild-type | wild-type | reduced | [22] |
| MGG_05153 | FZC73 | reduced | wild-type | wild-type | [1] |
| MGG_05247 | Mgd1 | wild-type | wild-type | reduced | [23] |
| MGG_05255 | ATG16 | wild-type | wild-type | 0 | [24] |
| MGG_05287 | CON7 | wild-type | reduced | 0 | [25] |
| MGG_05332 | MoPLC2 | reduced | reduced | reduced | [26] |
| MGG_05738 | PAX1 | reduced | 0 | reduced | [27] |
| MGG_06011 | MoSFA1 | reduced | reduced | reduced | [28] |
| MGG_06131 | Mobzip14 | reduced | reduced | reduced | [11] |
| MGG_06320 | CHM1 | reduced | reduced | 0 | [29] |
| MGG_06439 | Tea4 | reduced | redunced | 0 | [30] |
| MGG_06726 | MoSep4 | wild-type | wild-type | reduced | [31] |
| MGG_07173 | MoYpd1 | reduced | reduced | reduced | [32] |
| MGG_07335 | RPKA | reduced | reduced | reduced | [33] |
| MGG_07460 | MoHyR1 | wild-type | wild-type | reduced | [34] |
| MGG_07681 | FZC28 | reduced | wild-type | wild-type | [1] |
| MGG_08130 | FZC17 | wild-type | wild-type | wild-type | [1] |
| MGG_08212 | MoAtf1 | reduced | wild-type | reduced | [35] |
| MGG_08837 | SIZ1 | reduced | reduced | reduced | [36] |
| MGG_08850 | MoGti1 | wild-type | reduced | 0 | [37] |
| MGG_08896 | MoPEX11A | wild-type | wild-type | reduced | [38] |
| MGG_09027 | FZC49 | wild-type | wild-type | wild-type | [1] |
| MGG_09100 | CUT2 | reduced | reduced | reduced | [39] |
| MGG_09273 | FZC50 | reduced | wild-type | wild-type | [1] |
| MGG_09471 | NTH1 | wild-type | reduced | reduced | [40] |
| MGG_09559 | ATG9 | wild-type | reduced | 0 | [41] |
| MGG_09898 | MoMAC1 | reduced | reduced | reduced | [42] |
| MGG_10150 | MST50 | reduced | reduced | reduced | [43] |
| MGG_11764 | FZC55 | wild-type | wild-type | wild-type | [1] |
| MGG_12122 | MoGsk1 | reduced | reduced | reduced | [44] |
| MGG_14008 | MoRic8 | reduced | reduced | reduced | [45] |
| MGG_14728 | FZC48 | wild-type | wild-type | wild-type | [1] |
| MGG_14847 | MST11 | wild-type | reduced | 0 | [2] |
| MGG_14931 | VRF1 | increased | reduced | 0 | [46] |
| MGG_16444 | FZC22 | wild-type | reduced | wild-type | [1] |
| MGG_17060 | FZC24 | wild-type | wild-type | wild-type | [1] |
| MGG_17821 | FZC26 | wild-type | reduced | wild-type | [1] |
| MGG_00595 | MoSRE3 | reduced | wild-type | reduced | [47] |
| MGG_00672 | MoLeu3 | reduced | wild-type | reduced | [48] |
| MGG_11916 | LDP1 | wild-type | wild-type | reduced | [49] |
| MGG_04478 | MoFim1 | reduced | reduced | reduced | [50] |
| MGG_07964 | MoLAEA | wild-type | wild-type | reduced | [51] |
| MGG_03527 | MoUbp8 | reduced | reduced | reduced | [52] |

**References**

1. Lu J, Cao H, Zhang L, Huang P, Lin F. Systematic analysis of Zn2Cys6 transcription factors required for development and pathogenicity by high-throughput gene knockout in the rice blast fungus. PLoS Pathog. 2014; 10(10):e1004432. <https://doi.org/10.1371/journal.ppat.1004432> PMID: 25299517

2. Zhao X, Kim Y, Park G, Xu JR. A mitogen-activated protein kinase cascade regulating infection-related morphogenesis in *Magnaporthe grisea*. The Plant Cell. 2005; 17(4):1317-29. <https://doi.org/10.1105/tpc.104.029116> PMID: 15749760

3. Jeon J, Goh J, Yoo S, Chi MH, Choi J, Rho HS, et al. A putative MAP kinase kinase kinase, MCK1, is required for cell wall integrity and pathogenicity of the rice blast fungus, *Magnaporthe oryzae*. Mol Plant Microbe Interact. 2008; 21(5):525-34. <https://doi.org/10.1094/MPMI-21-5-0525> PMID: 18393612

4. Marroquin-Guzman M, Sun G, Wilson RA. Glucose-ABL1-TOR signaling modulates cell cycle tuning to control terminal appressorial cell differentiation. PLoS Genet. 2017; 13(1):e1006557. <https://doi.org/10.1371/journal.pgen.1006557> PMID: 28072818

5. Nguyen Q, Iritani A, Ohkita S, Vu BV, Yokoya K, Matsubara A, et al. A fungal Argonaute interferes with RNA interference. Nucleic Acids Res. 2018; 46(5):2495-508. <https://doi.org/10.1093/nar/gkx1301> PMID: 29309640

6. Chen Y, Le X, Sun Y, Li M, Zhang H, Tan X, et al. MoYcp4 is required for growth, conidiogenesis and pathogenicity in *Magnaporthe oryzae*. Mol Plant Pathol. 2017; 18(7):1001-11. <https://doi.org/10.1111/mpp.12455> PMID: 27377363

7. Pham KT, Inoue Y, Vu BV, Nguyen HH, Nakayashiki T, Ikeda K, et al. MoSET1 (Histone H3K4 methyltransferase in *Magnaporthe oryzae*) regulates global gene expression during infection-related morphogenesis. PLoS Genet. 2015; 11(7):e1005385. <https://doi.org/10.1371/journal.pgen.1005385> PMID: 26230995

8. Lim YJ, Kim KT, Lee YH. SUMOylation is required for fungal development and pathogenicity in the rice blast fungus *Magnaporthe oryzae*. Mol Plant Pathol. 2018; 19(9):2134-48. <https://doi.org/10.1111/mpp.12687> PMID: 29633464

9. Du Y, Shi Y, Yang J, Chen X, Xue M, Zhou W, et al. A serine/threonine-protein phosphatase PP2A catalytic subunit is essential for asexual development and plant infection in *Magnaporthe oryzae*. Curr Genet. 2013; 59(1-2):33-41. <https://doi.org/10.1007/s00294-012-0385-3> PMID: 23269362

10. Gupta YK, Dagdas YF, Martinez-Rocha AL, Kershaw MJ, Littlejohn GR, Ryder LS, et al. Septin-Dependent Assembly of the Exocyst Is Essential for Plant Infection by *Magnaporthe oryzae*. The Plant Cell. 2015; 27(11):3277-89. <https://doi.org/10.1105/tpc.15.00552> PMID: 26566920

11. Kong S, Park SY, Lee YH. Systematic characterization of the bZIP transcription factor gene family in the rice blast fungus, *Magnaporthe oryzae*. Environ Microbiol. 2015; 17(4):1425-43. <https://doi.org/10.1111/1462-2920.12633> PMID: 25314920

12. Weber RW, Wakley GE, Thines E, Talbot NJ. The vacuole as central element of the lytic system and sink for lipid droplets in maturing appressoria of *Magnaporthe grisea*. Protoplasma. 2001; 216(1-2):101-12. <https://doi.org/10.1007/BF02680137> PMID: 11732192

13. Chen J, Zheng W, Zheng S, Zhang D, Sang W, Chen X, et al. Rac1 is required for pathogenicity and Chm1-dependent conidiogenesis in rice fungal pathogen *Magnaporthe grisea*. PLoS Pathog. 2008; 4(11):e1000202. <https://doi.org/10.1371/journal.ppat.1000202> PMID: 19008945

14. Breth B, Odenbach D, Yemelin A, Schlinck N, Schroder M, Bode M, et al. The role of the Tra1p transcription factor of *Magnaporthe oryzae* in spore adhesion and pathogenic development. Fungal Genet Biol. 2013; 57:11-22. <https://doi.org/10.1016/j.fgb.2013.05.008> PMID: 23733043

15. Samalova M, Melida H, Vilaplana F, Bulone V, Soanes DM, Talbot NJ, et al. The beta-1,3-glucanosyltransferases (Gels) affect the structure of the rice blast fungal cell wall during appressorium-mediated plant infection. Cell Microbiol. 2017; 19(3):e12659. <https://doi.org/10.1111/cmi.12659> PMID: 27568483

16. Liu TB, Liu XH, Lu JP, Zhang L, Min H, Lin FC. The cysteine protease MoAtg4 interacts with MoAtg8 and is required for differentiation and pathogenesis in *Magnaporthe oryzae*. Autophagy. 2010; 6(1):74-85. <https://doi.org/10.4161/auto.6.1.10438> PMID: 19923912

17. Saunders DG, Dagdas YF, Talbot NJ. Spatial uncoupling of mitosis and cytokinesis during appressorium-mediated plant infection by the rice blast fungus *Magnaporthe oryzae*. The Plant Cell. 2010; 22(7):2417-28. <https://doi.org/10.1105/tpc.110.074492> PMID: 20639448

18. Wang J, Yin Z, Tang W, Cai X, Gao C, Zhang H, et al. The thioredoxin MoTrx2 protein mediates reactive oxygen species (ROS) balance and controls pathogenicity as a target of the transcription factor MoAP1 in *Magnaporthe oryzae*. Mol Plant Pathol. 2017; 18(9):1199-209. <https://doi.org/10.1111/mpp.12484>. PMID: 27560036

19. He M, Xu Y, Chen J, Luo Y, Lv Y, Su J, et al. MoSnt2-dependent deacetylation of histone H3 mediates MoTor-dependent autophagy and plant infection by the rice blast fungus *Magnaporthe oryzae*. Autophagy. 2018; 14(9):1543-61. <https://doi.org/10.1080/15548627.2018.1458171> PMID: 29929416

20. Yan X, Li Y, Yue X, Wang C, Que Y, Kong D, et al. Two novel transcriptional regulators are essential for infection-related morphogenesis and pathogenicity of the rice blast fungus *Magnaporthe oryzae*. PLoS Pathog. 2011; 7(12):e1002385. <https://doi.org/10.1371/journal.ppat.1002385> PMID: 22144889

21. Yin Z, Zhang X, Wang J, Yang L, Feng W, Chen C, et al. MoMip11, a MoRgs7-interacting protein, functions as a scaffolding protein to regulate cAMP signaling and pathogenicity in the rice blast fungus *Magnaporthe oryzae*. Environ Microbiol. 2018; 20(9):3168-85. <https://doi.org/10.1111/1462-2920.14102> PMID: 29727050

22. Wang ZY, Thornton CR, Kershaw MJ, Debao L, Talbot NJ. The glyoxylate cycle is required for temporal regulation of virulence by the plant pathogenic fungus *Magnaporthe grisea*. Mol Microbiol. 2003; 47(6):1601-12. <https://doi.org/10.1046/j.1365-2958.2003.03412.x> PMID: 12622815

23. Oh Y, Donofrio N, Pan H, Coughlan S, Brown DE, Meng S, et al. Transcriptome analysis reveals new insight into appressorium formation and function in the rice blast fungus *Magnaporthe oryzae*. Genome Biol. 2008; 9(5):R85. <https://doi.org/10.1186/gb-2008-9-5-r85> PMID: 18492280

24. Zhu XM, Li L, Wu M, Liang S, Shi HB, Liu XH, et al. Current opinions on autophagy in pathogenicity of fungi. Virulence. 2019; 10(1):481-9. <https://doi.org/10.1080/21505594.2018.1551011> PMID: 30475080

25. Odenbach D, Breth B, Thines E, Weber RW, Anke H, Foster AJ. The transcription factor Con7p is a central regulator of infection-related morphogenesis in the rice blast fungus *Magnaporthe grisea*. Mol Microbiol. 2007; 64(2):293-307. <https://doi.org/10.1111/j.1365-2958.2007.05643.x> PMID: 17378924

26. Choi J, Kim KS, Rho HS, Lee YH. Differential roles of the phospholipase C genes in fungal development and pathogenicity of *Magnaporthe oryzae*. Fungal Genet Biol. 2011; 48(4):445-55. <https://doi.org/10.1016/j.fgb.2011.01.001> PMID: 21237279

27. Li Y, Yue X, Que Y, Yan X, Ma Z, Talbot NJ, et al. Characterisation of four LIM protein-encoding genes involved in infection-related development and pathogenicity by the rice blast fungus *Magnaporthe oryzae*. Plos One. 2014; 9(2):e88246. <https://doi.org/10.1371/journal.pone.0088246> PMID: 24505448

28. Zhang Z, Wang J, Chai R, Qiu H, Jiang H, Mao X, et al. An S-(hydroxymethyl)glutathione dehydrogenase is involved in conidiation and full virulence in the rice blast fungus *Magnaporthe oryzae*. Plos One. 2015; 10(3):e0120627. <https://doi.org/10.1371/journal.pone.0120627> PMID: 25793615

29. Li L, Xue C, Bruno K, Nishimura M, Xu JR. Two PAK kinase genes, CHM1 and MST20, have distinct functions in *Magnaporthe grisea*. Mol Plant Microbe Interact. 2004; 17(5):547-56. <https://doi.org/10.1094/MPMI.2004.17.5.547> PMID: 15141959

30. Patkar RN, Suresh A, Naqvi NI. MoTea4-mediated polarized growth is essential for proper asexual development and pathogenesis in *Magnaporthe oryzae*. Eukaryot Cell. 2010; 9(7):1029-38. <https://doi.org/10.1128/EC.00292-09> PMID: 20472691

31. Feng HQ, Li GH, Du SW, Yang S, Li XQ, de Figueiredo P, et al. The septin protein Sep4 facilitates host infection by plant fungal pathogens via mediating initiation of infection structure formation. Environ Microbiol. 2017; 19(5):1730-49. <https://doi.org/10.1111/1462-2920.13613> PMID: 27878927

32. Jacob S, Foster AJ, Yemelin A, Thines E. High osmolarity glycerol (HOG) signalling in *Magnaporthe oryzae*: Identification of MoYPD1 and its role in osmoregulation, fungicide action, and pathogenicity. Fungal Biol. 2015; 119(7):580-94. <https://doi.org/10.1016/j.funbio.2015.03.003> PMID: 26058534

33. Selvaraj P, Tham HF, Ramanujam R, Naqvi NI. Subcellular compartmentation, interdependency and dynamics of the cyclic AMP-dependent PKA subunits during pathogenic differentiation in rice blast. Mol Microbiol. 2017; 105(3):484-504. <https://doi.org/10.1111/mmi.13713> PMID: 28544028.

34. Huang K, Czymmek KJ, Caplan JL, Sweigard JA, Donofrio NM. HYR1-mediated detoxification of reactive oxygen species is required for full virulence in the rice blast fungus. PLoS Pathog. 2011; 7(4):e1001335. <https://doi.org/10.1371/journal.ppat.1001335> PMID: 21533213

35. Guo M, Guo W, Chen Y, Dong S, Zhang X, Zhang H, et al. The basic leucine zipper transcription factor Moatf1 mediates oxidative stress responses and is necessary for full virulence of the rice blast fungus *Magnaporthe oryzae*. Mol Plant Microbe Interact. 2010; 23(8):1053-68. <https://doi.org/10.1094/MPMI-23-8-1053> PMID: 20615116

36. Liu C, Li Z, Xing J, Yang J, Wang Z, Zhang H, et al. Global analysis of sumoylation function reveals novel insights into development and appressorium-mediated infection of the rice blast fungus. New Phytol. 2018; 219(3):1031-47. <https://doi.org/10.1111/nph.15141> PMID: 29663402

37. Chen Y, Zhai S, Zhang H, Zuo R, Wang J, Guo M, et al. Shared and distinct functions of two Gti1/Pac2 family proteins in growth, morphogenesis and pathogenicity of *Magnaporthe oryzae*. Environ Microbiol. 2014; 16(3):788-801. <https://doi.org/10.1111/1462-2920.12204> PMID: 23895552

38. Wang J, Li L, Zhang Z, Qiu H, Li D, Fang Y, et al. One of Three Pex11 Family Members Is Required for Peroxisomal Proliferation and Full Virulence of the Rice Blast Fungus *Magnaporthe oryzae*. Plos One. 2015; 10(7):e0134249. <https://doi.org/10.1371/journal.pone.0134249> PMID: 26218097

39. Skamnioti P, Gurr SJ. *Magnaporthe grisea* cutinase2 mediates appressorium differentiation and host penetration and is required for full virulence. Plant Cell. 2007; 19(8):2674-89. <https://doi.org/10.1105/tpc.107.051219> PMID: 17704215

40. Foster AJ, Jenkinson JM, Talbot NJ. Trehalose synthesis and metabolism are required at different stages of plant infection by *Magnaporthe grisea*. Embo J. 2003; 22(2):225-35. <https://doi.org/10.1093/emboj/cdg018> PMID: 12514128

41. Dong B, Liu XH, Lu JP, Zhang FS, Gao HM, Wang HK, et al. MgAtg9 trafficking in *Magnaporthe oryzae*. Autophagy. 2009; 5(7):946-53. <https://doi.org/10.4161/auto.5.7.9161> PMID: 19556868.

42. Zhu L, Zhu J, Liu Z, Wang Z, Zhou C, Wang H. Host-Induced Gene Silencing of Rice Blast Fungus *Magnaporthe oryzae* Pathogenicity Genes Mediated by the Brome Mosaic Virus. Genes (Basel). 2017; 8(10). <https://doi.org/10.3390/genes8100241> PMID: 28954400

43. Li G, Zhang X, Tian H, Choi YE, Tao WA, Xu JR. MST50 is involved in multiple MAP kinase signaling pathways in *Magnaporthe oryzae*. Environ Microbiol. 2017; 19(5):1959-74. <https://doi.org/10.1111/1462-2920.13710> PMID: 28244240

44. Zhou T, Dagdas YF, Zhu X, Zheng S, Chen L, Cartwright Z, et al. The glycogen synthase kinase MoGsk1, regulated by Mps1 MAP kinase, is required for fungal development and pathogenicity in *Magnaporthe oryzae*. Sci Rep. 2017; 7(1):945. <https://doi.org/10.1038/s41598-017-01006-w> PMID: 28424497

45. Li Y, Yan X, Wang H, Liang S, Ma WB, Fang MY, et al. MoRic8 Is a novel component of G-protein signaling during plant infection by the rice blast fungus *Magnaporthe oryzae*. Mol Plant Microbe Interact. 2010; 23(3):317-31. <https://doi.org/10.1094/MPMI-23-3-0317> PMID: 20121453

46. Cao H, Huang P, Zhang L, Shi Y, Sun D, Yan Y, et al. Characterization of 47 Cys2 -His2 zinc finger proteins required for the development and pathogenicity of the rice blast fungus *Magnaporthe oryzae*. New Phytol. 2016; 211(3):1035-51. <https://doi.org/10.1111/nph.13948> PMID: 27041000

47. Chung H, Kim S, Kim KT, Hwang BG, Kim HJ, Lee SJ, et al. A novel approach to investigate hypoxic microenvironment during rice colonization by *Magnaporthe oryzae*. Environ Microbiol. 2019; 21(3):1151-69. <https://doi.org/10.1111/1462-2920.14563> PMID: 30773773

48. Wei YY, Yu Q, Dong B, Zhang Y, Liu XH, Lin FC, et al. MoLEU1, MoLEU2, and MoLEU4 regulated by MoLEU3 are involved in leucine biosynthesis, fungal development, and pathogenicity in *Magnaporthe oryzae*. Environ Microbiol Rep. 2019; 11(6):784-96. <https://doi.org/10.1111/1758-2229.12800> PMID: 31621205

49. Cai X, Yan J, Liu C, Xing J, Ren Z, Hendy A, et al. Perilipin LDP1 coordinates lipid droplets formation and utilization for appressorium-mediated infection in *Magnaporthe oryzae*. Environ Microbiol. 2020; 22(7):2843-57. <https://doi.org/10.1111/1462-2920.15019> PMID: 32291878

50. Li YB, Xu R, Liu C, Shen N, Han LB, Tang D. *Magnaporthe oryzae* fimbrin organizes actin networks in the hyphal tip during polar growth and pathogenesis. PLoS Pathog. 2020; 16(3):e1008437. <https://doi.org/10.1371/journal.ppat.1008437> PMID: 32176741

51. Saha P, Ghosh S, Roy-Barman S. MoLAEA Regulates Secondary Metabolism in *Magnaporthe oryzae*. mSphere. 2020; 5(2): e00936-19. <https://doi.org/10.1128/mSphere.00936-19> PMID: 32238572

52. Yang J, Chen D, Matar KAO, Zheng T, Zhao Q, Xie Y, et al. The deubiquitinating enzyme MoUbp8 is required for infection-related development, pathogenicity, and carbon catabolite repression in *Magnaporthe oryzae*. Appl Microbiol Biotechnol. 2020; 104(11):5081-94. <https://doi.org/10.1007/s00253-020-10572-5> PMID: 32274561
