# Supplementary material for: The rice blast fungus SR protein 1 regulates alternative splicing with unique mechanisms
Source: PLoS Pathog. 2022 Dec 8;18(12):e1011036. doi: 10.1371/journal.ppat.1011036 (PMC9767378; doi:10.1371/journal.ppat.1011036)
Supplement: S5 Table — (DOCX) [file ppat.1011036.s014.docx]

**S5 Table. Fungal strains used in this study.**

| **Strain** | **Description** | **References** |
| --- | --- | --- |
| P131 | A wild-type strain | [1] |
| srp1ko1 | A deletion mutant of *MoSRP1* | This study |
| cSRP1 | srp1ko1 expressing the wild-type *MoSRP1* allele | This study |
| cSRP1^△1-90^ | srp1ko1 expressing the MoSRP1^△1-90^ allele | This study |
| cSRP1^△91-206^ | srp1ko1 expressing the MoSRP1^△91-206^ allele | This study |
| M117 | srp1ko1 expressing the MoSRP1^S117A^ allele | This study |
| M119 | srp1ko1 expressing the MoSRP1^S119A^ allele | This study |
| M193 | srp1ko1 expressing the MoSRP1^S193A^ allele | This study |
| M117/119 | srp1ko1 expressing the MoSRP1^S117A/S119A^ allele | This study |
| M117/119/M193 | srp1ko1 expressing the MoSRP1^S117A/S119A/S193A^ allele | This study |
| Srp1-flag-3 | srp1ko1 expressing the wild-type *MoSRP1* allele with 3Flag | This study |
| Srp1-flag-7 | srp1ko1 expressing the wild-type *MoSRP1* allele with 3Flag | This study |
| RP27-flag-1 | P131 expressing the 3Flag tag empty vector | This study |
| RP27-flag-1 | P131 expressing the 3Flag tag empty vector | This study |
| Δ*Moatf1* | A deletion mutant of *MoATF1* | [2] |
| MoATF1/Δ*Moatf1* | Δ*Moatf1* expressing the *MoATF1* allele | This study |
| MoATF1^CAAC^/Δ*Moatf1*  Δ*Momtp1*  MoMTP1/Δ*Momtp1*  MoMTP1^CAAC^/Δ*Momtp1* | Δ*Moatf1* expressing the MoATF1^CAAC^ allele  A deletion mutant of *MoMTP1*  Δ*Momtp1* expressing the *MoMTP1* allele  Δ*Momtp1* expressing the MoMTP1^CAAC^ allele | This study  This study  This study  This study |
| smt3ko1/MoSrp1-GFP | smt3ko1 expressing the MoSrp1-GFP allele | This study |
| srp1ko1/MoSrp1^K78R/E80Q^-GFP | smt3ko1 expressing the MoSrp1^K78R/E80Q^-GFP allele | This study |
| FgSrp1/srp1ko1 | srp1ko1 expressing the FgSrp1 allele | This study |
| AtRBP1/srp1ko1 | srp1ko1 expressing the AtRBP1 allele | This study |
| AtSC35/srp1ko1 | srp1ko1 expressing the AtSC35allele | This study |
| SpSRP1/srp1ko1 | srp1ko1 expressing the SpSRP1 allele | This study |
| SpSRP2/srp1ko1 | srp1ko1 expressing the SpSRP2 allele | This study |
| MoSrp1^1-130^ | srp1ko1 expressing the MoSrp1^1-130^ allele | This study |
| SpSrp1^1-130^ | srp1ko1 expressing the SpSrp1^1-130^ allele | This study |
| MoSrp1^1-60^+SpSrp1^61-130^ | srp1ko1 expressing the MoSrp1^1-60^ and SpSrp1^61-130^ allele | This study |
| SpSrp1^1-60^+MoSrp1^61-130^ | srp1ko1 expressing the SpSrp1^1-60^ and MoSrp1^61-130^ allele | This study |
| SpSrp1^80K81I^ | srp1ko1 expressing the SpSrp1^80K81I^ allele | This study |

**References**

1. Peng YL, Shishiyama J. Temporal sequence of cytological events in rice leaves infected with *Pyricularia oryzae*. Can J Bot.1988; 66: 730-735.57.
2. Guo M, Guo W, Chen Y, Dong S, Zhang X, Zhang H, et al. The basic leucine zipper transcription factor Moatf1 mediates oxidative stress responses and is necessary for full virulence of the rice blast fungus *Magnaporthe oryzae*. Mol Plant Microbe Interact. 2010; 23(8):1053-68. <https://doi.org/10.1094/MPMI-23-8-1053> PMID: 20615116
